# Therapeutic effect of baicalin on experimental autoimmune encephalomyelitis is mediated by SOCS3 regulatory pathway

**DOI:** 10.1038/srep17407

**Published:** 2015-11-30

**Authors:** Yuan Zhang, Xing Li, Bogoljub Ciric, Cun-Gen Ma, Bruno Gran, Abdolmohamad Rostami, Guang-Xian Zhang

**Affiliations:** 1 Department of Neurology, Thomas Jefferson University, Philadelphia, PA, USA; 2 Key Laboratory of the Ministry of Education for Medicinal Resources and Natural Pharmaceutical Chemistry, Northwest China National Engineering Laboratory for Resource Development of Endangered Crude Drugs, College of Life Sciences, Shaanxi Normal University, Xi’an, China; 3 Institute of Brain Science, Department of Neurology, Shanxi Datong University Medical School, Datong, China; 4 Clinical Neurology Research Group, Division of Clinical Neuroscience, University of Nottingham School of Medicine, UK

## Abstract

Natural compounds derived from medicinal plants have long been considered a rich source of novel therapeutic agents. Baicalin (Ba) is a bioactive flavonoid compound derived from the root of *Scutellaria baicalensis,* an herb widely used in traditional medicine for the treatment of various inflammatory diseases. In this study, we investigate the effects and mechanism of action of Ba in experimental autoimmune encephalomyelitis (EAE), an animal model of multiple sclerosis (MS). Ba treatment effectively ameliorated clinical disease severity in myelin oligodendrocyte glycoprotein (MOG)35–55 peptide-induced EAE, and reduced inflammation and demyelination of the central nervous system (CNS). Ba reduced infiltration of immune cells into the CNS, inhibited expression of proinflammatory molecules and chemokines, and prevented Th1 and Th17 cell differentiation via STAT/NFκB signaling pathways. Further, we showed that SOCS3 induction is essential to the effects of Ba, given that the inhibitory effect of Ba on pathogenic Th17 responses was largely abolished when SOCS3 signaling was knocked down. Taken together, our findings demonstrate that Ba has significant potential as a novel anti-inflammatory agent for therapy of autoimmune diseases such as MS.

Multiple sclerosis (MS) and its animal model, experimental autoimmune encephalomyelitis (EAE), are inflammatory demyelinating diseases of the central nervous system (CNS)^1^,^2^. EAE can be induced by immunization with myelin antigens or adoptive transfer of myelin-specific CD4 + T cells that mediate the destruction of myelin and neural axons^3^. Based on distinctive cytokine secretion and transcription factor expression, effector CD4 + T cells are classified in at least four major subsets, i.e., Th1, Th2, Th17, and regulatory T (Treg) cells^4^,^5^. Complex cytokine networks are critical for determining CD4 + T cell fate and, in general, more than one cytokine is required for differentiation to any particular subset^6^. For Th1 differentiation, IL-12 and IFN-γ are two important cytokines. Many cytokines including IL-4, IL-2, and IL-7 may be involved in Th2 differentiation. While TGF-β promotes Th17 differentiation in the presence of IL-6, it also induces Treg cell differentiation in the presence of IL-2 and absence of IL-6^5^,^6^,^7^.

While the precise contribution of each Th subset to autoimmunity is still debated, it is generally accepted that Th1 and Th17 cells are pro-inflammatory subsets responsible for inducing autoimmune and inflammatory processes, while Tregs have protective and anti-inflammatory effects^6^,^7^,^8^. Currently approved therapies for MS either have limited efficacy or pose significant safety concerns^1^. Thus, discovering new drugs that specifically target pathogenic Th1 and Th17 cells, while sparing other immune cells, is important for the development of more effective MS treatment. Recently, research exploring novel anti-inflammatory or immunomodulatory drugs derived from medicinal plants has attracted a great deal of attention^9^,^10^. These plants represent a rich source of natural compounds for the identification of safe and effective candidate drugs with novel targets and/or mechanism of action in the treatment of autoimmune diseases.

*Scutellaria baicalensis* is a well-known medicinal plant that has been widely used in Asia for centuries in the treatment of inflammation, allergy, and bacterial and viral infections^11^,^12^, but whose mechanism of action remains undefined. Baicalin (Ba), a bioactive flavonoid compound derived from the root of *S. baicalensis*, is thought to be the major component responsible for the biological effects of this medicinal plant^12^. Ba exhibits anti-inflammatory, anti-tumor, anti-oxidant, anti-apoptotic, anti-viral, and antibacterial properties^13^,^14^,^15^,^16^,^17^, indicating its potential as a treatment for autoimmune diseases. Preliminary evidence has linked these properties of Ba to inducing inflammatory cell apoptosis, promoting Treg cell differentiation and regulatory activity, inhibiting T-cell proliferation, suppressing IFN-γ and increasing IL-4 production^18^,^19^,^20^. Although these results are encouraging, more direct evidence is needed on the beneficial effect and molecular mechanisms of Ba in autoimmunity. In particular, the effect of Ba on the immunopathogenesis of autoimmune inflammatory diseases, e.g., pro-inflammatory action of Th17 cells, has not been studied. To address these questions, in the present study we used the EAE model to investigate the potential therapeutic effects and underlying mechanism of Ba in inflammatory/autoimmune disorders. The treatment efficacy of Ba in EAE made it possible to identify the cell types and signal pathways involved in the regulatory actions of Ba.

## Results

### Ba effectively ameliorates clinical EAE.

**Figure 1:**
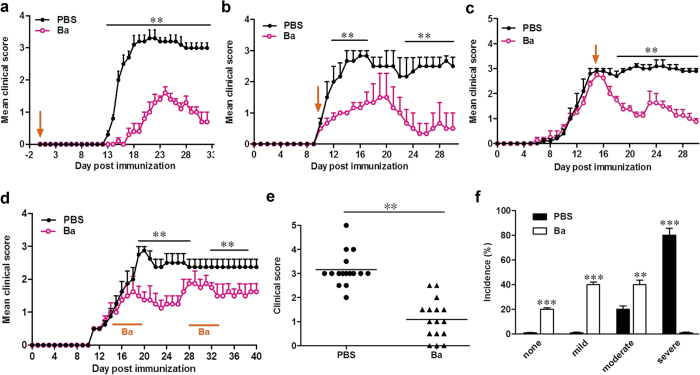
Ba Ameliorates Clinical Symptoms of EAE. C57BL/6 mice were injected i.p. with PBS (●) or Ba (100 mg/kg, ○) daily (**a**) at the day of EAE induction, (**b**) day 10 p.i. (disease onset), (**c**) day 15 p.i. (disease peak), or (**d**) during the indicated time points. Results are shown as mean ± SEM (*n* = 5 each group). (**e**) Disease distribution at the end points of experiment, and (**f)** incidence of disease severity at the end points of experiment when treatment started at day 10 p.i. Disease severity is graded as severe (clinical score: > 3), moderate (clinical score: 1.5–3), mild (clinical score: <1.5) or none (no clinical signs). One representative of three independent experiments is shown in a, b, and c. The data in d, e, and f came from three independent experiments when Ba was injected from day 10 p.i. (n = 5–6 each group). **P < 0.01; ***P < 0.001.

To determine the anti-inflammatory properties of Ba, we examined its effects in EAE. By testing different doses, we found that Ba at 100 mg/kg/d is optimal for suppressing EAE (Fig. s1); this dose was therefore chosen for all subsequent *in vivo* experiments. For the prophylactic treatment regimen, Ba administration starting from day 0 post immunization (p.i.) resulted in delayed onset and significantly decreased disease severity compared to PBS-treated control mice (*P* < 0.01; Fig. 1a and Table s1). In the therapeutic regimen, Ba administration starting from disease onset (day 10 p.i.) or its peak (day 15 p.i.) effectively suppressed EAE progression (Fig. 1b,c). Improvement in clinical signs was observed 1–2 days after Ba injection, and the effect persisted until the end of the experiment. To investigate the lasting effect of Ba administration, treatment was started from disease onset (day 14 p.i.) and stopped when disease development was significantly suppressed by Ba (average clinical score <1.5, day 19 p.i.). Recurrence of disease signs was observed 7–8 days after stopping Ba treatment, while re-exposure of these mice to the same treatment of Ba resulted in a significant suppression of clinical signs (Fig. 1d), suggesting the necessity for continuous daily use. Together, Ba treatment substantially reduced disease severity (Fig. 1e,f and Table s1); the majority of Ba-treated EAE mice displayed mild or moderate signs, while severe disease was only observed in PBS-treated mice (Fig. 1f). These results suggest that Ba has a significant therapeutic effect in EAE.

### Ba Treatment Reduces CNS Inflammation.

**Figure 2:**
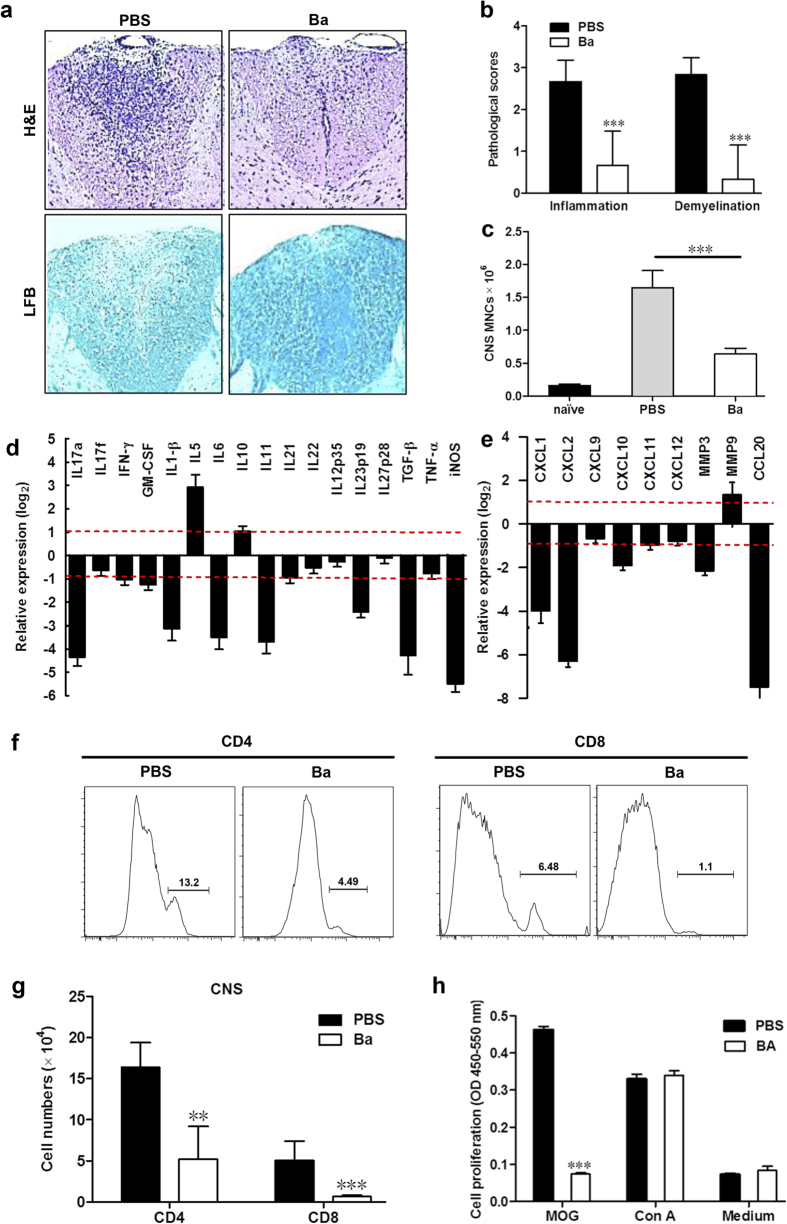
Ba Treatment Reduced CNS Inflammation. Ba- or PBS-treated control EAE mice described in Fig. 1c (treatment protocol) were sacrificed at day 30 p.i., and spinal cords were harvested. Sections at lumbar level (L3) were analyzed (**a**) by H&E (for inflammation; left) and Luxol fast blue (LFB) (for degree of demyelination; right), and (**b**) pathology scores of inflammation and demyelination are expressed as mean ± SD (*n* = 6 each group). The absolute numbers of MNCs in the CNS of above mice were counted (*n* = 3 each group) (**c**), and expression of cytokine (**d**) and chemokine (**e**) genes was determined using real-time RT-PCR analysis, and their relative expression was calculated by log_2_ of −ΔΔCt values from triplicate of PCR. More than two fold changes (log_2_ < −1 or log_2_ > 1) were considered significant between groups (red dotted line). (**f**) The percentage of CD4^+^ or CD8^+^ in the lymphocyte gate of the CNS of the above mice was analyzed by flow cytometry. (**g**) Absolute numbers of CD4^+^ or CD8^+^ cells in spinal cord were calculated (*n* = 5 each group). (**h**) Splenocytes of Ba- or PBS-treated EAE mice were isolated, stimulated with MOG_35–55_ (25 μg/ml) or Con A (5 μg/ml) and examined for proliferation at 72 h culture using BrdU incorporation assay (*n* = 6 each group). Data are expressed as mean ± SEM. **P < 0.01; ***P < 0.001. One representative of three experiments is shown.

To assess the effects of Ba on EAE-associated CNS pathology, lumbar spinal cords were obtained from Ba-treated and control mice. Analysis of CNS tissue sections demonstrated significantly reduced inflammation and demyelination in Ba-treated animals (Fig. 2a, right) when compared with control group (Fig. 2a, left). The pathological scores of mice treated with PBS or Ba differed significantly (*P* = 0.031 for the infiltration score and *P* = 0.022 for the demyelination score) (Fig. 2b). The number of mononuclear cells (MNCs) isolated from the CNS of Ba-treated mice was markedly reduced compared with that of vehicle-treated mice (*P* = 0.016; Fig. 2c).

To determine how Ba administration inhibits inflammatory cell infiltration, we assayed cytokine/chemokine gene expression in the spinal cords of PBS- and Ba-treated EAE mice. As shown in Fig. 2d,e, expression of inflammatory cytokines, including IL-17 A, IFN-γ, GM-CSF, IL1-β, IL-6, IL-1 and IL-23, was substantially reduced in mice treated with Ba (Fig. 2d). Ba treatment also inhibited production of TGF-β (Fig. 2d), a cytokine that plays roles in induction of Tregs^21^ and Th17 cells^22^. It has been reported that inducible nitric oxide synthase (iNOS) plays various roles in EAE^23^. We found elevated levels of iNOS in the lumbar spinal cord of mice with EAE that were markedly reduced by Ba treatment (Fig. 2d). Expression of matrix metalloproteinase MMP3 and virtually all the chemokines we tested, including those induced by IFN-γ signaling (CXCL-9, CXCL-10 and CXCL11) and by IL-17 signaling (CXCL1, CXCL2, CCL20)^24^, was significantly reduced (Fig. 2e). Ba treatment resulted in significantly decreased percentages and absolute numbers of CD4^+^ and CD8 T cells in the CNS as determined by flow cytometry (*P* < 0.01) (Fig. 2f,g). However, the percentages of these cells in the spleen remained unaltered (Fig. s2). In addition, splenocytes of Ba-treated mice showed a lower proliferative response to MOG35–55 peptide than those of PBS-treated mice (*P* < 0.001), while proliferation in response to a non-specific stimulus concanavalin A (Con A) did not differ (Fig. 2h). The reason for this phenomenon could be that, while MOG-specific T cells were activated and proliferated during EAE progression, this was significantly suppressed by Ba treatment. Therefore, the starting percentage and absolute number of MOG-specific T cells in Ba-treated mice were remarkably lower than in PBS controls in *ex vivo* cultures, resulting in a lower proliferation response to MOG35–55 peptide. By contrast, non-MOG-specific T cells, the majority of the T cell population, were in a resting state in EAE mice, and were thus not affected by Ba treatment; consequently, these cells retained the same normal proliferation response to Con A *ex vivo* as those from the PBS-treated group. This notion is also supported by our observation that Ba treatment in naïve B6 mice did not influence their T cell proliferative response to Con A when compared to PBS-treated mice (Fig. s7b).

### Th1 and Th17 Cell Subsets were Selectively Suppressed by Ba Treatment via the STAT and NF-κB Signaling Pathways.

**Figure 3:**
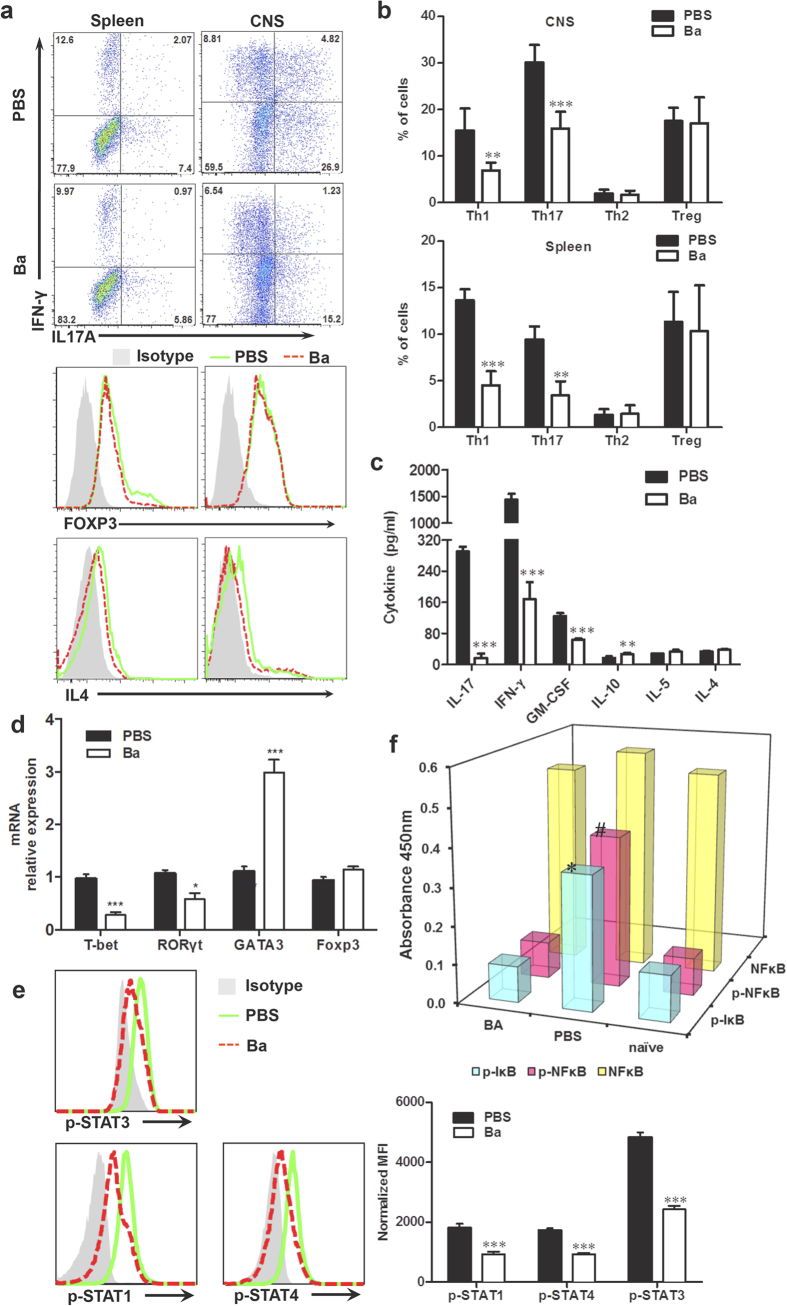
Th1 and Th17 cell subsets are selectively reduced by Ba via STAT and NF-κB signaling pathways. EAE mice were treated with Ba or PBS starting at day 10 p.i. and splenocytes or CNS MNCs from these mice were harvested at day 18 p.i. (**a**) Subsets of Th1, Th17, Th2, and Treg cells in CD4^+^ gate were analyzed by intracellular staining of IFN-γ, IL-17, IL-4, and Foxp3, following stimulation with MOG_35–55_ (25 μg/ml) for 72 h for spleen or with MOG_35–55_ (10 μg/ml) for 24 h for CNS cells. (**b**) Percentages of cells positive for these cytokines in CNS (up) and spleen (down) are expressed as mean ± SEM (n = 3 each group). (**c**) Supernatants derived from splenocyte cultures described in (**a**) were analyzed for the level of indicated cytokines (mean ± SEM; n = 6 each group). (**d**) mRNA levels of T-bet, RORγt, GATA3, and Foxp3 from spleens of EAE mice treated with Ba or PBS were analyzed by real-time PCR. (**e**) Splenocytes were cultured in the presence of MOG_35–55_ (25 μg/ml) for 72 h and analyzed by intracellular staining for phosphorylation level of indicated STAT proteins. (**f**) Supernatants from cell cultures in (**c**) were analyzed for phosphorylated IκBα, NF-κB p65, and phosphorylated NF-κB p65 by PathScan Inflammation Multi-Target Sandwich ELISA Kit (n = 6 each group). Data are expressed as mean ± SEM. #, *P < 0.05; **P < 0.01; ***P < 0.001. One representative of three experiments is shown.

The decreased numbers of CD4^+^ T cells in the CNS of mice treated with Ba prompted us to investigate which subsets among these cells were affected. Ba significantly reduced the percentages of MOG-reactive Th1 (CD4^+^IFN-γ^+^) and Th17 (CD4^+^ IL-17^+^) cells, both in the spleen (*P* = 0.0005 and *P* = 0.0067, respectively) and the CNS (*P* = 0.0028 and *P* = 0.0006, respectively) compared with the vehicle control, while numbers of Th2 (CD4^+^IL4^+^) and Treg (CD4^+^Foxp3^+^) cells did not differ (Fig. 3a,b). Consistent with this, production of IFN-γ and IL-17 was significantly reduced by Ba treatment (*P* < 0.001, Fig. 3c). We also showed that GM-CSF, a critical cytokine in Th17 cell pathogenicity^24^, was significantly decreased by Ba treatment (*P* < 0.001, Fig. 3c). In contrast, low but detectable levels of the Th2 cytokines IL-4 and IL-5 were not affected by Ba (Fig. 3c). Anti-inflammatory cytokine IL-10 showed only a small, but significant, increase (Fig. 3c). We also defined the effect of Ba on cytokine production of splenocytes from untreated EAE mice *in vitro*. Ba significantly suppressed IFN-γ, IL-17 and GM-CSF, and up-regulated IL-10 production *in vitro* (Fig. s3).

In parallel, expression of key transcription factors T-bet for Th1 cells and ROR-γt for Th17 cells, but not Foxp3 for Treg, was significantly reduced in splenocytes derived from Ba-treated EAE mice (Fig. 3d). Interestingly, expression of GATA3, the master regulator for the Th2 subset, was increased significantly (*P* < 0.001, Fig. 3d). These results suggest that Ba suppresses EAE by halting Th1 and Th17 cell development, and may induce immunoregulatory cytokines. Because STAT transcription factors play a crucial role in the differentiation of Th cells^25^, we hypothesized that Ba exerted regulatory effects on Th1 and Th17 development through direct inhibition of a STAT pathway. To this end, splenocytes isolated from Ba- or PBS-treated EAE mice were cultured in the presence of MOG_35–55_ for 72 h, and then analyzed for phosphorylation of the key STAT molecules. As shown in Fig. 3e, consistent with the reduction in numbers of Th1 and Th17 cells, levels of phosphorylated (p)-STAT1 and p-STAT4 for Th1 cells and p-STAT3 for Th17 cells were decreased.

We further addressed the effect of Ba treatment on antigen presenting cells, e.g., dendritic cells (DCs) and macrophages/microglia. To this end, splenocytes and CNS MNCs of Ba- or PBS-treated EAE mice were isolated at day 18 p.i. and expression of co-stimulatory molecules CD80 and CD86 on CD11b^+^ (macrophages/microglia) and CD11c^+^ (DCs) cells was analyzed by flow cytometry. Results demonstrated that, while Ba- and PBS-treated mice had similar percentages of CD11b^+^ cells in the CNS and spleen, a reduced percentage of CD11c^+^ DCs was observed in the Ba-treated group compared to the PBS-treated group. Further, DCs and macrophages/microglia in the Ba-treated group expressed reduced levels of CD80 and CD86 in both CNS MNCs and splenocytes compared to PBS-treated mice (Fig. s4). These results suggest that Ba inhibited APC activation during EAE development.

The NF-κB pathway has been implicated in the pathogenesis of autoimmune diseases^26^, and the fact that Ba downregulated NF-κB activation in kidney cells^27^ led us to hypothesize that Ba acts mainly through immune-regulating mechanisms. We found that, while there was basal phosphorylation of NF-κB p65 and IκB-α in splenocytes of naïve control mice, significantly elevated phosphorylation of NF-κB p65 (3.9 ± 0.3-fold, P < 0.01) and IκB-α (2.8 ± 0.1-fold, P < 0.01) was present in spleen of vehicle-treated EAE mice. In contrast, in BA-treated EAE mice, phosphorylation of NF-κBp65 and IκB-α was significantly decreased to 49.2 ± 5.7% and 66.9 ± 7.5% (P < 0.01), respectively, compared with control EAE mice, while total NF-κB expression was not affected (Fig. 3f).

Taken together, these data suggest that the therapeutic effect of Ba results from a selective inhibition of STAT and NF-κB pathways *in vivo*.

### Ba inhibits encephalitogenicity of Th cells in Adoptive EAE

**Figure 4:**
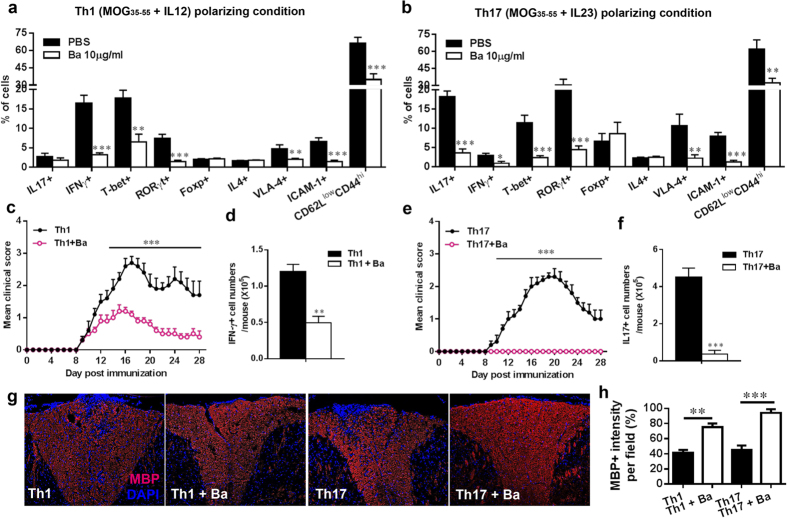
Ba inhibited the capacity of Th1/Th17 cells to induce adoptive EAE. For adoptive transfer EAE, single-cell suspensions were derived from spleen and lymph nodes of EAE mice at day 10 p.i. MOG (25 μg/ml) plus IL-12 (10 ng/ml) or IL-23 (10 ng/ml) were added to cultures in the presence or absence of Ba (10 μg/ml) for 3 days. The resulting cells were analyzed by flow cytometry under (**a**) Th1 (MOG_35–55_ + IL-12) or (**b**) Th17 (MOG_35–55_ + IL-23) polarizing conditions. Cultured T cells mentioned above were i.v. injected into naïve female C57BL/6 mice, 8–10 weeks of age, at 3 × 10^7^ Th1 cells per mouse (**c**) or 1.5 × 10^7^ Th17 cells per mouse (**e**). Mice were observed daily for EAE severity. At day 18 post injection, CNS MNCs from these mice were harvested and analyzed by flow cytometry. Absolute numbers of IFN-γ^+^ (**d**) or IL-17^+^ (**f**) cells were calculated by multiplying the total numbers of MNCs and percentage of IFN-γ^+^ or IL-17^+^ cells in CD4^+^ gate. Lumbar spinal cords were isolated for immunohistochemistry. (**g**) MBP immunohistochemistry on spinal cord sections of mice adoptively transferred Th1/Th17 cells mentioned above. (**h**) Quantitative analysis of MBP intensity measured at random areas in the white matter of spinal cords using Imagepro. Data are expressed as mean ± SEM (n = 5 each group). *P < 0.05; **P < 0.01; ***P < 0.001. One representative of three experiments is shown.

To test the effect of Ba on the pathogenicity of Th1 and Th17 cells, we prepared single-cell suspensions from spleen and lymph nodes of mice that had been immunized with MOG35–55 seven days earlier and cultured them under Th1 (MOG_35–55_ +IL-12) or Th17 (MOG_35–55_ +IL-23) polarizing conditions in the presence or absence of Ba for 3 days. The resulting cells were analyzed for the percentage of T cell subsets, expression of transcription factor, adhesion molecules and activation markers by flow cytometry. Ba treatment significantly reduced numbers of IFN-γ- and IL-17-producting cells under Th1 and Th17 polarizing conditions, respectively (*P* < 0.001), while the numbers of Th2 (CD4^+^IL-4^+^) and Treg (CD4^+^ Foxp3^+^) cells did not differ (Fig. 4a,b). Consistent with this observation, expression of key transcription factors T-bet for Th1 cells and ROR-γt for Th17 cells, but not Foxp3 for Treg, was significantly decreased in the presence of Ba under Th1 or Th17 polarizing conditions (*P* < 0.01, Fig. 4a,b). Surface epitopes such as adhesion molecules (VLA-4 and ICAM-1) and T cell activation markers (CD62L^low^CD44^hi^) were also significantly inhibited under Ba treatment (Fig. 4a,b). These results indicated that Ba inhibits encephalitogenicity of Th cells by suppressing production of pro-inflammatory cytokines, crucial adhesion molecule expression and T cell activation.

Then we transferred the cultured T cells mentioned above into naïve recipient mice to evaluate the pathogenic activates. As shown in Fig. 4c,e, clinical EAE was developed in the recipient mice having received T cells that had not been exposed to Ba. Ba treatment completely abolished the encephalitogenicity of Th17 cells (Fig. 4e), and significantly inhibited encephalitogenicity of Th1 cells (Fig. 4c). Consistent with clinical observations, Ba treatment significantly suppressed inflammatory infiltration of Th1 (CD4^+^IFN-γ^+^, Fig. 4d) or Th17 (CD4^+^IL-17^+^, Fig. 4f) cells into the CNS of recipients (*P* < 0.01 and *P* < 0.001, respectively). To further evaluate the extent of demyelination, spinal cords were collected from adoptive transfer recipients at disease peak, and MBP expression was examined by immunohistochemistry. As shown in Fig. 4g,h, demyelinated lesions were detected by reduced MBP density in the white matter of the lumbar region of spinal cords of untreated Th1/Th17 cell-induced EAE mice, whereas mice injected with Ba-treated Th1/Th17 cells exhibited much smaller demyelinated areas. Similar results were observed in sections from the brain stem region (data not shown). All together, these data demonstrate that Ba effectively inhibited encephalitogenicity of Th1 and Th17 cells in adoptive EAE, with a more profound effect on Th17 cells.

### Ba exerts Therapeutic Effects on EAE by Suppressing Th1 and Th17 Cell Development

To further elucidate the mechanism underlying the effect of Ba on CD4^+^ T cells, we characterized its effect on Th1 and Th17 differentiation and proliferation *in vitro*. We first measured viability and apoptosis of T cells in different Ba concentrations in order to find the optimal conditions for *in vitro* experiments (Fig. s5). Up to 20 μg/ml, an effective concentration, Ba did not affect cell viability, which was decreased in concentrations ≥50 μg/ml of Ba (Fig. s5a). Also, 5-20 μg/ml concentrations did not significantly increase the percentage of apoptotic cells (Fig. s5b), indicating that Ba lacks cytotoxicity toward CD4^+^ T cells at concentrations up to 20 μg/ml. We then measured CD4^+^ T cell proliferation under Ba treatment with the BrdU incorporation test (Fig. s5c). Ba suppressed CD4^+^ T cell proliferation in a dose-dependent manner (5–20 μg/ml). Together, these results show that the observed suppression of CD4^+^ T cell proliferation by Ba is not due to cell death.

**Figure 5:**
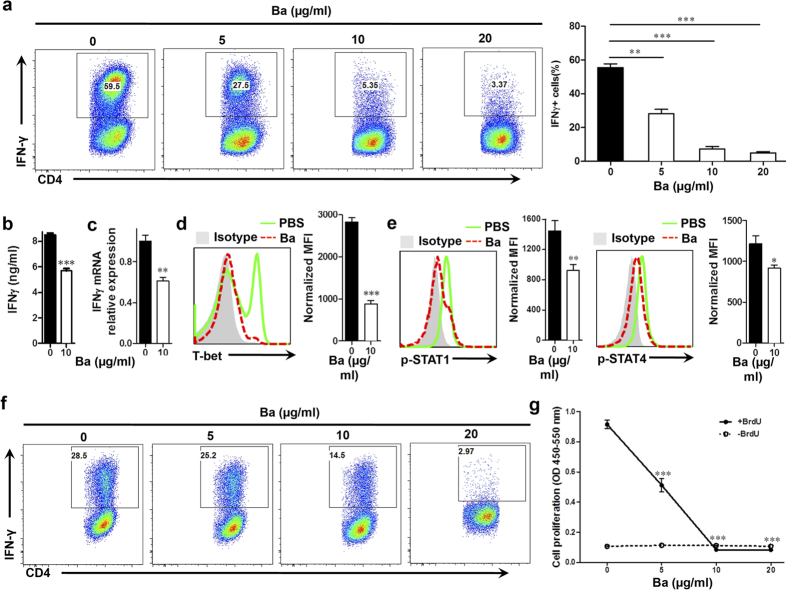
Role of Ba in Th1 cell differentiation and proliferation. Purified naïve CD4^+^ T cells were cultured with different concentrations of Ba under Th1 polarizing conditions and analyzed at 3 days of culture. (**a**) The percentage of Th1 cells in CD4^+^ T cells was analyzed by intracellular IFN-γ secretion. (**b**) IFN-γ production in culture supernatants was analyzed by ELISA. (**c**) IFN-γ mRNA levels were analyzed by real-time PCR. Intracellular levels of T-bet (**d**) and phosphorylation of STAT1 and STAT4 (**e**) of CD4^+^ T cells was analyzed using flow cytometry. (**f**) The above-mentioned differentiated Th1 cells were rested, washed and cultured for a second stimulation with IL-12 in the presence of Ba. Percentage of Th1 cells was analyzed by intracellular staining of IFN-γ. (**g**) Proliferation of pre-differentiated Th1 cell was measured by BrdU incorporation assay. The same cell preparations as in (**f**) were labeled with BrdU for 24 h of culture. Data are expressed as mean ± SEM (n = 5 each group). **P < 0.01; ***P < 0.001. One representative of three experiments is shown.

Under Th1-polarizing conditions, approximately 60% of CD4^+^ cells were IFN-γ^+^ in the PBS-control group, and Ba treatment significantly inhibited Th1 cell differentiation in a dose-dependent manner (Fig. 5a). While the presence of Ba at a dose of 5 μg/ml during differentiation reduced Th1-polarized (IFN-γ-producing) CD4^+^ T cells by one half (25.4 ± 2.45% vs. 62.3 ± 4.75% in control, *P* < 0.01), Th1 cell differentiation was more effectively suppressed at higher doses of Ba, e.g., 10–20 μg/ml (6.36 ± 2.22% and 2.85 ± 1.14%, respectively, both *P* < 0.001). In agreement with this, IFN-γ production and expression were suppressed by Ba (Fig. 5b, c), which correlated with decreased T-bet expression (Fig. 5d), as well as decreased STAT1 and STAT4 phosphorylation (Fig. 5e). These results suggest that Ba hinders Th1 differentiation by inhibiting its key players, T-bet, STAT1 and STAT4. We also investigated the effect of Ba on already differentiated Th1 cells. Ba inhibited CD4^+^ IFNγ^+^ cells in a dose-dependent manner (Fig. 5f), and reduced their proliferation (Fig. 5g).

**Figure 6:**
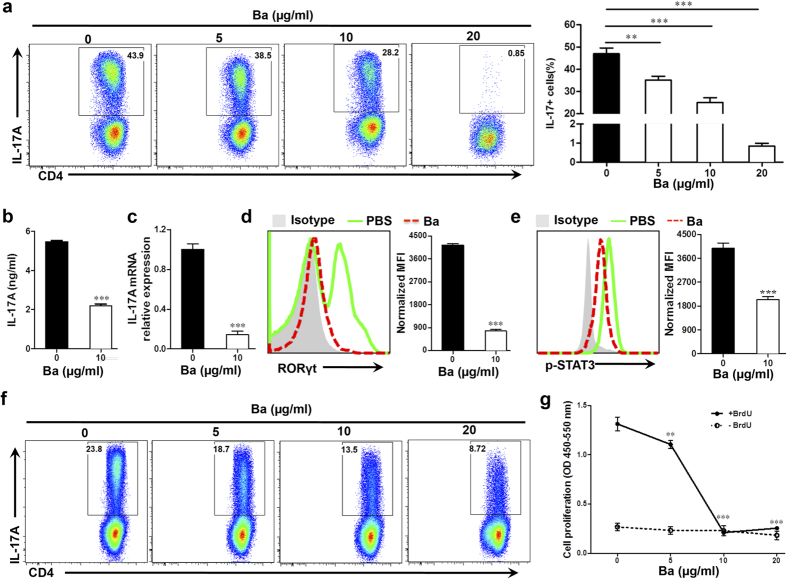
Role of Ba in Th17 cell differentiation and proliferation. Naïve CD4^+^ cells were cultured with different concentrations of Ba under the Th17 polarizing condition at 3 days of culture. (**a**) Percentage of Th17 cells was analyzed by intracellular staining of IL-17. (**b**) Culture supernatants were analyzed for IL-17 production. (**c**) *IL17a* mRNA levels were analyzed by real-time PCR. Intracellular levels of RORγt (**d**) and phosphorylation of STAT3 (**e**) were analyzed using flow cytometry. (**f**) The above-mentioned differentiated Th17 cells were rested, washed and cultured for a second stimulation with IL-23 in the presence of Ba. Percentage of Th17 cells was analyzed by intracellular staining of IL-17. (**g**) Proliferation of pre-differentiated Th17 cell was measured by BrdU incorporation assay. The same cell preparations as in F were labeled with BrdU and cultured for 24 h. Data are expressed as mean ± SEM. (n = 5 each group). **P < 0.01; ***P < 0.001. One representative of three experiments is shown.

Ba also significantly inhibited Th17 cell differentiation (Fig. 6a) and reduced IL-17 A production and expression in a dose-dependent manner (5–20 μg/ml) (Fig. 6b,c). RORγt is the master regulator for Th17 differentiation, and STAT3 is the key transducer of IL-6, IL-21, and IL-23 signaling^28^,^29^. Consistent with its effect on Th17 differentiation, Ba suppressed RORγt expression (Fig. 6d) as well as its upstream event, STAT3 phosphorylation (Fig. 6e), under Th17 polarizing condition. Further, we found that addition of Ba into already differentiated Th17 cells reduced the percentage and proliferation of CD4^+^ IL-17^+^ cells in a dose-dependent manner (Fig. 6f,g). These results, together with those shown in Fig. 5, indicate that Ba suppresses the differentiation and proliferation of both Th1 and Th17 cells.

Given that Ba treatment *in vivo* induced expression of GATA3, the key transcription factor for Th2 (Fig. 3d), we then determined the *in vitro* effect of Ba on Th2 differentiation. Under a Th2-polarizing condition, approximately 40% of CD4^+^ cells were IL-4^+^ in the PBS-treated group, and this was mildly, but significantly, increased by Ba treatment in a dose-dependent manner (*P* < 0.05, Fig. S6a,b). These data suggested a shift toward an anti-inflammatory phenotype by Ba. Consistently, expression of GATA3 was also significantly increased in the presence of Ba in a dose-dependent manner under Th2 polarizing condition (*P* < 0.001, Fig. s6c).

### Ba Suppresses Th17 Cell Development by Up-Regulating Expression of SOCS3

**Figure 7:**
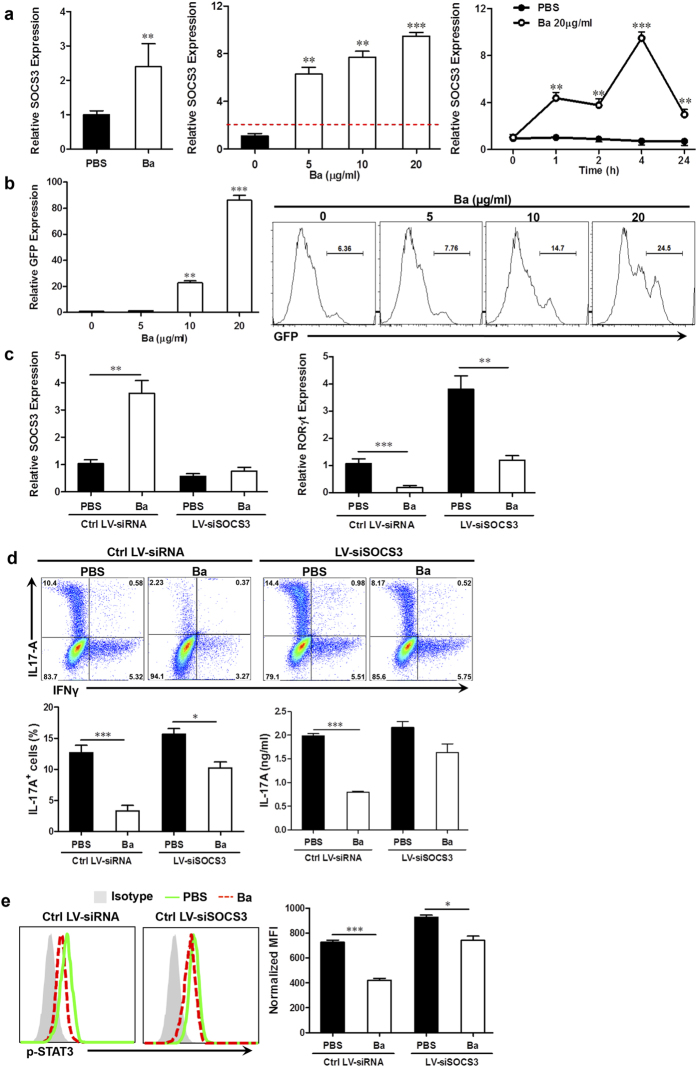
Induction of SOCS3 underlies inhibition of Th17 cell differentiation by Ba. (**a**) SOCS3 expression was determined by real-time PCR in CD4^+^ T cells from spleens of Ba- or PBS-treated EAE mice (left), or in naïve CD4^+^ cells under Th17 cell differentiation conditions at different concentrations of Ba (middle) and harvested at time points indicated, with a concentration of Ba at 20 μg/ml (right). (**b**) SOCS3 promoter activity was determined in CD4^+^ T cells transfected with a 1619-bp murine SOCS3 promoter-GFP reporter in the presence of Ba (5–20 μg/ml). GFP expression and production were determined by RT-PCR and flow cytometry. (**c**) SOCS3 (left) and RORγt (right) expression in CD4^+^ T cells transfected with SOCS3-specific or control LV-siRNAs under Th17 cell polarization conditions in the presence or absence of Ba (20 μg/ml). IL-17 A production (**d**) and STAT3 phosphorylation (**e**) were measured by flow cytometry and ELISA in CD4^+^ T cells cultured in conditions described in **C**. Data are expressed as mean ± SEM. (n = 5). *P < 0.05; **P < 0.01; ***P < 0.001. One representative of three experiments is shown.

Next, we investigated the upstream signaling events involved in the alteration of Th1/Th17 responses by Ba. Given that suppressor of cytokine signaling 3 (SOCS3) plays an important role in inhibiting Th17 response^30^ as well as Th1 response^31^, we tested if SOCS3 plays a role in the effects of Ba. Indeed, upon Ba treatment, SOCS3 expression was increased in CD4^+^ T cells in Ba-treated EAE mice both *in vivo* (Fig. 7a, left) and *in vitro* (Fig. 7a, middle and right). The basal level of SOCS3 expression was detected during Th17 polarization, while its expression was significantly elevated by Ba in a dose-dependent manner (approximately 6~10-fold higher). Upon 20 μg/ml treatment, SOCS3 expression was up-regulated as early as 1 h, continued to peak at 4 h and remained elevated for at least 24 h.

To further confirm that Ba induces SOCS3 expression, CD4^+^ T cells were transfected with a 1619-bp long murine SOCS3 promoter-GFP reporter and treated with Ba at different doses. As shown in Fig. 7b, Ba produced a robust (approximately tenfold) induction of SOCS3 promoter activity in a dose dependent manner. Further, while Ba treatment induced SOCS3 expression, this capacity was diminished when SOCS3-siRNA (siSOCS3) was added to the culture (Fig. 7c left), confirming the success of SOCS3 knockdown.

The causal relationship between Ba-induced SOCS3 expression and the inhibition of Th17 cell development was verified by knockdown of SOCS3 expression with lentivirus-delivered siRNA (LV-siRNAs). As shown in Fig. 7c (right), SOCS3 knockdown resulted in high RORγt expression in CD4^+^ T cells, and adding Ba to these cells inhibited this expression, which remained at a comparable level to cells treated with PBS and control siRNA. Interestingly, Ba-induced Th17 suppression was largely abolished by blocking expression of SOCS3 (Fig. 7d). It was evident that, when SOCS3 was knocked down in CD4^+^ T cells, Ba treatment had little effect on RORγt expression (Fig. 7c), Th17 cell differentiation (Fig. 7d), and STAT3 phosphorylation (Fig. 7e), confirming a SOCS3-dependent mechanism underlying the inhibition of Th17 cell differentiation by Ba. In conclusion, our data demonstrate that Ba significantly increased expression of SOCS3, which subsequently prevented the phosphorylation of STAT3, resulting in decreased development of Th17 cells.

### Safety consideration of Ba treatment

Ba has been safely used for treatment in several animal models of diseases, e.g., collagen-induced arthritis in rats^11^, murine adjuvant-induced arthritis^32^, murine model of polymicrobial sepsis^13^, rat model of Alzheimer’s disease^33^ and renal ischemia-reperfusion injury in rats^27^. Further, pharmacokinetic properties of Ba have been well investigated^34^. Both oral administration and i.p. injection were used for Ba treatment at a wide range of dosage (10~800 mg/kg/day) in various animal models^11^,^13^,^27^,^32^,^33^,^35^,^36^,^37^,^38^. Due to baicalin’s poor solubility in water, its absolute bioavailability after oral administration is only 2.2%^39^. Therefore, in the present study, we chose i.p. injection daily with the dosage of 100 mg/kg/day based on our dose optimization study.

Naïve female C57BL/6 mice (6–8 weeks old) were injected i.p. daily with PBS or Ba at 100 mg/kg/day, the same dosage as for treating EAE mice, and the administration continued for 28 d. Body weights of mice were recorded and blood was analyzed for red blood cell count, hemoglobin concentration, white blood cell count and platelet count by the automated hematologic analyzer after the last administration. For *ex vivo* proliferation, splenocytes were isolated from PBS- and Ba-treated mice, and stimulated with or without Con A (5 μg/ml). Ba treatment did not lead to weight loss (Fig. s7a), total number of splenocytes and T cell proliferative response to Con A (Fig. s7b), or to major hematopoietic changes (Fig. s7c), indicating an acceptable safety of this compound *in vivo*.

## Discussion

Natural compounds derived from medicinal plants have long been considered a rich source of novel therapeutic agents. *Scutellaria baicalensis,* of which Ba is the major active compound^12^, has been used as an anti-inflammatory drug in traditional herbal medicine and has recently shown a good safety record^11^,^12^. We in the present study demonstrate that Ba effectively suppressed EAE via SOCS3-induced inhibition of Th1 and Th17 cell differentiation. At effective doses, Ba treatment only moderately inhibited T cell proliferation, without discernible effects on T cell viability or apoptosis *in vitro*, and minimally affected the entire T cell response *in vivo*. These results highlight the potential of Ba as an immunomodulatory agent with minimal side effects.

One important limitation in using natural product derivatives to treat disease is our often limited knowledge of their mechanisms of action, which adds to our misgivings about clinical use. While it has been suggested that Ba suppresses development of EAE in SJL/J mice and rats^18^,^20^ by inducing IL-4 and inhibiting IFN-γ^18^, as well as by promoting apoptosis of inflammatory cells in the spinal cord^20^, the mechanism of Ba action in the differentiation and function of various Th cell subsets in autoimmune disease has not yet been described. In the present study we demonstrate that Ba can regulate differentiation and activity of Th17 and Th1 cells without affecting Th2 and regulatory T cells. This was first demonstrated by diminished Th1/Th17 response in EAE mice treated with Ba and further substantiated by adoptive-transfer experiments showing the loss of encephalitogenicity in Ba-treated, MOG-specific Th17 cells. The STAT signaling pathway is known to be a major signaling network involved in Th cell differentiation^28^,^29^,^40^. We found that Ba selectively acts on pathogenic Th1 and Th17 cells, while sparing Th2 and Treg cells. Different Th lineages rely on distinct signaling pathway(s) for their development, providing a mechanistic basis for differential effects of Ba on various Th lineages. In this way, Ba inhibited STAT3 phosphorylation and RORγt expression in differentiating Th17 cells and reduced STAT4 and STAT1 phosphorylation and T-bet expression during Th1 differentiation. To our knowledge, this is the first demonstration that Ba regulates T cell differentiation and function through the STAT pathway.

Furthermore, our study provides a detailed account of the underlying molecular mechanism and the key signaling events responsible for the inhibition of Th17 cell differentiation by Ba. Our *in vitro* and *in vivo* studies show that the SOCS3-STAT3 pathway is the key axis that mediates inhibition of Th17 cell differentiation in response to Ba treatment, as upregulation of SOCS3 by Ba results in reduced STAT3 activation and Th17 cell differentiation. The role of SOCS3 described here is consistent with previous reports indicating that SOCS3 deficiency in T cells results in higher numbers of Th17 cells both *in vitro* and *in vivo*^30^,^41^, and that transduction of SOCS3 in DCs inhibits Th17 cell differentiation and, subsequently, suppresses EAE^42^. Although our *in vitro* study did not show an effect of SOCS3-knockdown on IFN-γ production, it has been reported that systemic delivery of adenovirus encoding SOCS3 prevented development of collagen-induced arthritis, and SOCS3-transfected APCs significantly suppressed IFN-γ production by T cells in culture, indicating an inhibitory effect on Th1 cells^31^. Whether SOCS3 plays a negative regulatory role in Th1 cell differentiation, as it does in Th17 cells, is worthy of further investigation.

Although MS and EAE pathogenesis depends on activation of CD4^+^ T cells, innate immune cells also play important roles in disease progression. Among them, dendritic cells (DCs) as professional APCs have a potent capacity to prime naïve T cells and activate autoreactive response^43^. It has been shown that Ba impairs Th1 polarization through inhibition of DC maturation , and inhibits expression of surface molecules CD80, CD86, MHC class I, and MHC class II as well as the levels of IL-12 production in lipopolysaccharide-stimulated DCs^44^. Similar inhibitory effects in Ba-treated EAE mice were also observed in our study. In addition to the direct effects on DC and Th cells, Ba administration modified the cytokine microenvironment, which governs the differentiation and activation of Th cells. This effect may be mediated, at least in part, by inhibition of the NF-κB signaling pathway, which plays an essential role during Th1 and Th17 development. We found that Ba significantly inhibited phosphorylation of NF-κB p65 and IκB-α, a result that is consistent with previous studies^11^.

It is known that pathogenesis of autoimmune diseases involves the breakdown of multiple regulatory pathways^9^,^10^. Thus, anti-inflammatory drugs obtained from a single, target-based drug discovery process may be unlikely to achieve adequate efficacy in the treatment of autoimmune conditions. In comparison, multiple compounds from traditional herbal medicine that target distinct immune/inflammation pathways may be more successful at reining in the immune system^9^,^10^. The regulatory effects of Ba on the immune response include suppression of DC maturation, inhibition of Th1/Th17 cell development and proliferation, and re-ordering the cytokine microenvironment. The powerful therapeutic effects of natural compounds have motivated search for new drugs^45^. For example, fingolimod (FTY720), the first oral treatment for MS, which was approved in 2010^46^, was discovered by chemical modification of a natural product, myriocin^47^, which is isolated from *Cordyceps sinensis*, a fungus used in Chinese traditional medicine^48^. Likewise, the novel anti-inflammatory properties of Ba that we discovered through mechanistic studies in EAE show its potential for the development of pathway-based immunomodulatory therapeutics.

## Materials and Methods

### EAE Induction and Treatment

Female C57BL/6 mice, 8–10 weeks of age, were purchased from the Jackson Laboratory (Bar Harbor, ME). All experimental procedures and protocols were approved by the Institutional Animal Care and Committee of Thomas Jefferson University and were carried out in accordance with the approved institutional guidelines and regulations. Mice were immunized s.c. for active induction of EAE as described^49^. Briefly, for active EAE, mice were immunized subcutaneously on the back with 200 μg of myelin oligodendrocyte glycoprotein (MOG) 35–55 peptide (MEVGWYRSPFSRVVHLYRNGK) emulsified in CFA (Difco Lab, Detroit, MI) containing 4 mg/ml *Mycobacterium tuberculosis* H37Ra (Difco). Two hundred nanograms of pertussis toxin (List Biological Lab, Epsom, England) were given intraperitoneally on days 0 and 2 post-immunization (p.i.). Clinical scores were calculated blindly by two researchers daily according to a 0–5 scale as follows^50^: 1, limp tail or waddling gait with tail tonicity; 2, waddling gait with limp tail (ataxia); 2.5, ataxia with partial limb paralysis; 3, full paralysis of 1 limb; 3.5, full paralysis of one limb with partial paralysis of the second limb; 4, full paralysis of two limbs; 4.5, moribund; and 5, death. Ba was obtained from Sigma-Aldrich (St. Louis, MO). The resulting compound used in this study had a purity of 95%; it was dissolved in DMSO to provide stock solution and was diluted before injection with PBS to the final concentration. Dosage was determined based on a dose optimization study (Fig. s1). Ba (100 mg/kg/day) was injected intraperitoneally (i.p.) daily starting on the day of immunization (prevention protocol), 10 days p.i. (disease onset) or 15 days p.i. (disease peak).

For adoptive transfer EAE, single-cell suspensions were derived from spleen and lymph nodes of EAE mice (day 10 p.i.). MOG_35–55_ (25 μg/ml) plus IL-12 (10 ng/ml) or IL-23 (10 ng/ml) were added to induce proliferation of MOG-reactive Th1 or Th17 cells in the presence or absence of Ba (10 μg/ml) for 3 days, and analyzed by flow cytometry. The resulting cells (3 × 10^7^ Th1 cells per mouse and 1.5 × 10^7^ Th17 cells per mouse) were administered i.v. into recipients. The recipient mice also received pertussis toxin (200 ng/mouse) i.v. on days 0 and 2 post transfer. Mice were examined daily and scored for disease severity using the standard scale^51^,^52^.

### Histopathology

For CNS histopathological assessment, mice were perfused with PBS transcardially, and then with 4% paraformaldehyde. Tissues were treated with ethanol and xylene, and paraffin-embedded 5 μm sections were stained with H&E for assessment of inflammation and with Luxol fast blue (LFB) for demyelination. Slides were assessed in a blinded fashion for inflammation and demyelination using a 0–3 scale as described^50^. For immunohistochemistry, brainstem and spinal cord tissues were fixed using 4% paraformaldehyde for 1 day and then cryo-protected using 30% sucrose solution for 3 days. Fixed tissues were embedded in OCT compound (Tissue-Tek, Sakura Finetek, Japan) for frozen sections and then sectioned coronally at 12 μm. Transverse sections of tissues were cut and stained with different primary antibodies. Immunofluorescence controls were routinely performed with slides in which primary antibodies were not included. Results were visualized by fluorescent microscopy (Nikon Eclipse E600; Nikon, Melville, NY).

### Preparation of Infiltrating MNCs in CNS

For preparation of infiltrating mononuclear cells (MNCs) from spinal cord and brain (hereafter referred to as CNS), mice were perfused with 30 ml PBS via the heart to eliminate peripheral blood. Single-cell suspensions were prepared, MNCs were harvested using a Percoll (Sigma-Aldrich, St. Louis, MO) gradient (70/37%), and viable cells were counted in 0.4% Trypan blue.

### T cell proliferation and cytokine measurement

For *ex vivo* proliferation, splenocytes were isolated 18 days p.i. from vehicle-treated and Ba-treated mice, and examined for proliferation with or without stimuli (25 μg/ml MOG_35–55_ or 5 μg/ml concanavalin A). For *in vitro* assay, purified CD4^+^ T cells from spleens of C57BL/6 mice were stimulated with anti-CD3e (5 μg/ml) and anti-CD28 (2 μg/ml) in different concentrations of Ba (5–20 μg/ml) for 24 h for cell viability assay and 72 h for cell proliferation and apoptosis assays. T cell viability was measured using a MTS kit (CellTiter 96^®^ AQueous One Solution Cell Proliferation Assay System Protocol, Promega). Cell apoptosis was measured using a FITC Annexin V Apoptosis Detection Kit I (BD Biosciences). Cell proliferation was determined by BrdU-incorporation test using BrdU Cell Proliferation ELISA Kit (Abcam). Supernatants from splenocytes or CD4^+^ T cells prepared as above were collected at 72 h to measure concentrations of IFN-γ, IL-17, IL-5, IL-10, and GM-CSF using ELISA kits (R&D Systems, Minneapolis, MN). Levels of phosphorylated IκBα, NF-κB p65, and phosphorylated NF-κB p65 in splenocytes of Ba-or vehicle-treated mice were analyzed by PathScan^®^ Inflammation Multi-Target Sandwich ELISA Kit (Cell Signaling Technology Inc.) following the manufacturer’s instructions.

### Flow cytometry

For surface-marker staining, cells were incubated with fluorochrome-conjugated Abs to CD4, CD8, CD11b, CD11c, CD80 CD86, VLA-4, ICAM-1, CD62L and CD44 (BD Biosciences, San Jose, CA) at the recommended dilution or isotype control Abs for 30 min on ice. To analyze MOG-specific Th1, Th2 and Th17 cells, splenocytes or CNS-infiltrating MNCs were stimulated with 25 μg/ml MOG peptide for 72 h or overnight, followed by stimulation with 50 ng/ml PMA and 500 ng/ml ionomycin in the presence of GolgiPlug for 5 h. Cells were surface-stained with mAbs against CD4 and CD8. Cells were then washed, fixed, and permeabilized with Fix & Perm Medium (Invitrogen), and intracellular cytokines were stained with Abs against IL17, IFN-γ, or IL4 (BD Biosciences). For phosphorylated STAT staining, cells were fixed with 4% paraformaldehyde for 10 min at 37 °C, permeabilized with 90% methanol for 30 min on ice, and stained with p-STAT1, p-STAT3, p-STAT4 and CD4 mAbs (BD Biosciences, San Jose, CA). Foxp3 staining was carried out using a commercial kit, according to the manufacturer’s instructions (eBioscience, San Diego, CA). Flow cytometric analysis was performed on FACSAria (BD Biosciences, San Jose, CA) and data were analyzed with FlowJo software (Treestar, Ashland, OR).

### *In vitro* CD4 + T cell polarization and proliferation

Differentiation of Th1, Th2 and Th17 cells was induced *in vitro* following protocols previously described^9^,^10^. Briefly, single-cell suspensions derived from spleen of normal female C57BL/6 mice (6–8 wk) were purified by negative selection with a mouse CD4^+^ T Cell Isolation Kit II (Miltenyi Biotec). Purified naïve CD4^+^ T cells were cultured for 3 days with soluble anti-CD3e (5 μg/ml) and anti-CD28 (2 μg/mL) under their respective polarizing conditions. IL-12 (5 ng/ml) was added to induce differentiation into Th1 cells. anti-IFN-γ (10 μg/ml), IL-2 (10 ng/ml), and IL-4 (30 ng/ml) were added to induce T cell differentiation into Th2 cells. TGF-β1 (2 ng/ml), IL-6 (20 ng/ml), IL-1β (10 ng/ml), anti-IL-4 (10 μg/ml), and anti-IFN-γ (10 μg/ml) were added in Th17 polarizing conditions. For the proliferation of pre-differentiated Th1 or Th17 cells, cells were rested 2 days in the presence of IL-2 (2 ng/ml), washed and replated for a second stimulation with anti-CD3e (5 μg/ml) and anti-CD28 (2 μg/ml) in the presence either of IL-23 (10 ng/ml) or medium. Cells were cultured for 3 days and labeled with BrdU for cell proliferation assay by an ELISA reader following the manufacturer’s instructions (R&D Systems, Minneapolis, MN).

### Quantitative real-time PCR

Total RNA was isolated from cell pellets using RNeasy Mini Kit (Qiagen, Valencia, CA), and first-strand cDNA was subsequently synthesized using Sensiscript RT Kit (Qiagen) according to the manufacturer’s instructions. mRNA expression was determined by real-time PCR using SYBR Green Master Mix under standard thermocycler conditions (Applied Biosystems, Foster City, CA). Data were collected and quantitatively analyzed on an ABI Prism 7900 Sequence Detection System (Applied Biosystems). Mouse *GAPDH* gene was used as endogenous control for sample normalization. Results were presented as fold increases relative to the expression of mouse *GAPDH*. Sequences of PCR primers are listed in Table S2.

### Vector construction and transduction

To determine SOCS3 promoter activity, a 1036-bp (from −107 to +929) minimal murine SOCS3 promoter was cloned and fused to the coding region of GFP as reporter (SOCS3-GFP). To knock down SOCS3 expression, vectors expressing SOCS3-shRNA were designed and synthesized, which consisted of a BamHI site, a 19-nucleotide sense sequence, a loop sequence, a 19-nucleotide antisense sequence, a RNA polymerase site, and an EcoRI site. The forward and reverse strand oligo sequences are as follows (the SOCS3 sense and antisense sequences are bolded, italics indicate loop): siSOCS3 Forward: 5′- GATCC**AGAGCAAAGAAAGGGTCAG***TTCAAGAGA***CTGACCCTTTCTTTGCTCTTTTTT**G -3′; siSOCS3 Reverse: 5′-AATTC**AAAAAAGAGCAAAGAAAGGGTCAG*****TCTCTTGAA*****CTGACCCTTTCTTTGCTCT**G-3′. The following complementary oligonucleotide was used as a negative control oligo sequence: Ctrl forward: 5′-GATCC**TTCTCCGAACGTGTCACGT***TTCAAGAGA***ACGTGACACGTTCGGAGAATTTTT**G-3′: Ctrl reverse: 5′-AATTC**AAAAATTCTCCGAACGTGTCACGT*****TCTCTTGAA*****ACGTGACACGTTCGGAGAA**G-3′. The forward and reverse strand oligo sequences were annealed to create double stranded oligonucleotides, which were then cloned into the pSIH1-H1-copGFP vector. Viruses were produced according to the user’s manual (SBI, CA). For cell infection, 5 × 10^5^ IU/mL virus and 8 μg/ml polybrene (Millipore, MA) were incubated with cells. After overnight incubation, the medium was replaced by fresh medium, and cultured for future use. For direct *in vivo* injection, approximately 3 × 10^7^ IU of recombinant lentivirus was injected i.c.v. into each mouse.

### Statistics

The Student’s t-test was used to analyze differences between groups. Where appropriate, one-way ANOVA was initially performed to determine whether an overall statistically significant change existed before a two-tailed paired or unpaired Student’s t-test was carried out. A *P* value < 0.05 was considered statistically significant.

## Additional Information

**How to cite this article**: Zhang, Y. *et al.* Therapeutic effect of baicalin on experimental autoimmune encephalomyelitis is mediated by SOCS3 regulatory pathway. *Sci. Rep.* **5**, 17407; doi: 10.1038/srep17407 (2015).

## Supplementary Material

Supplementary Information

## References

[b1] Thompson A. J., Toosy A. T. & Ciccarelli O. Pharmacological management of symptoms in multiple sclerosis: current approaches and future directions. Lancet Neurol 9, 1182–99 (2010). 21087742 10.1016/S1474-4422(10)70249-0

[b2] Peterson L. K. & Fujinami R. S. Inflammation, demyelination, neurodegeneration and neuroprotection in the pathogenesis of multiple sclerosis. J Neuroimmunol 184, 37–44 (2007). 17196667 10.1016/j.jneuroim.2006.11.015 PMC1933528

[b3] Rao P. & Segal B. M. Experimental autoimmune encephalomyelitis. Methods Mol Biol 900, 363–80 (2012). 22933079 10.1007/978-1-60761-720-4_18

[b4] Cosmi L. *et al.* T helper cells plasticity in inflammation. Cytometry A 85, 36–42 (2014). 24009159 10.1002/cyto.a.22348

[b5] Geginat J. *et al.* Plasticity of human CD4 T cell subsets. Front Immunol 5, 630 (2014). 25566245 10.3389/fimmu.2014.00630 PMC4267263

[b6] Christie D. & Zhu J. Transcriptional regulatory networks for CD4 T cell differentiation. Curr Top Microbiol Immunol 381, 125–72 (2014). 24839135 10.1007/82_2014_372 PMC4556129

[b7] Song X., Gao H. & Qian Y. Th17 differentiation and their pro-inflammation function. Adv Exp Med Biol 841, 99–151 (2014). 25261206 10.1007/978-94-017-9487-9_5

[b8] Stromnes I. M. *et al.* Differential regulation of central nervous system autoimmunity by T(H)1 and T(H)17 cells. Nature medicine 14, 337–42 (2008). 10.1038/nm1715 PMC2813727 18278054

[b9] Qin X. *et al.* Regulation of Th1 and Th17 cell differentiation and amelioration of experimental autoimmune encephalomyelitis by natural product compound berberine. Journal of immunology (Baltimore, Md: 1950) 185, 1855–63 (2010). 10.4049/jimmunol.0903853 20622114

[b10] Lu Y. *et al.* Eriocalyxin B ameliorates experimental autoimmune encephalomyelitis by suppressing Th1 and Th17 cells. Proc Natl Acad Sci USA 110, 2258–63 (2013). 23345445 10.1073/pnas.1222426110 PMC3568304

[b11] Wang H. Z. *et al.* Inhibitory effect of baicalin on collagen-induced arthritis in rats through the nuclear factor-kappaB pathway. The Journal of pharmacology and experimental therapeutics 350, 435–43 (2014). 24893986 10.1124/jpet.114.215145

[b12] Li-Weber M. New therapeutic aspects of flavones: the anticancer properties of Scutellaria and its main active constituents Wogonin, Baicalein and Baicalin. Cancer Treat Rev 35, 57–68 (2009). 19004559 10.1016/j.ctrv.2008.09.005

[b13] Zhu J. *et al.* Baicalin improves survival in a murine model of polymicrobial sepsis via suppressing inflammatory response and lymphocyte apoptosis. PloS one 7, e35523 (2012). 22590504 10.1371/journal.pone.0035523 PMC3348138

[b14] Nayak M. K. *et al.* Antiviral activity of baicalin against influenza virus H1N1-pdm09 is due to modulation of NS1-mediated cellular innate immune responses. J Antimicrob Chemother 69, 1298–310 (2014). 24458510 10.1093/jac/dkt534

[b15] Zheng W. X. *et al.* Baicalin protects PC-12 cells from oxidative stress induced by hydrogen peroxide via anti-apoptotic effects. Brain Inj 28, 227–34 (2014). 24456060 10.3109/02699052.2013.860469

[b16] Kong F. *et al.* Baicalin protects the myocardium from reperfusion-induced damage in isolated rat hearts via the antioxidant and paracrine effect. Exp Ther Med 7, 254–9 (2014). 24348801 10.3892/etm.2013.1369 PMC3861453

[b17] Wang S. C. *et al.* Baicalin scavenges reactive oxygen species and protects human keratinocytes against UVC-induced cytotoxicity. In Vivo 27, 707–14 (2013). 24292572

[b18] Zeng Y. *et al.* Baicalin reduces the severity of experimental autoimmune encephalomyelitis. Braz J Med Biol Res 40, 1003–10 (2007). 17653455 10.1590/s0100-879x2006005000115

[b19] Yang J., Yang X. & Li M. Baicalin, a natural compound, promotes regulatory T cell differentiation. BMC complementary and alternative medicine 12, 64 (2012). 22591709 10.1186/1472-6882-12-64 PMC3479077

[b20] Xu J. *et al.* [Effects of baicalin on apoptosis in rats with autoimmune encephalomyelitis]. Zhongguo Dang Dai Er Ke Za Zhi 13, 665–8 (2011). 21849120

[b21] Zamarron B. F. & Chen W. Dual roles of immune cells and their factors in cancer development and progression. Int J Biol Sci 7, 651–8 (2011). 21647333 10.7150/ijbs.7.651 PMC3107473

[b22] Ivanov I. I., Zhou L. & Littman D. R. Transcriptional regulation of Th17 cell differentiation. Semin Immunol 19, 409–17 (2007). 18053739 10.1016/j.smim.2007.10.011 PMC2696342

[b23] Farias A. S. *et al.* Nitric oxide and TNFalpha effects in experimental autoimmune encephalomyelitis demyelination. Neuroimmunomodulation 14, 32–8 (2007). 17700038 10.1159/000107286

[b24] El-Behi M. *et al.* The encephalitogenicity of T(H)17 cells is dependent on IL-1- and IL-23-induced production of the cytokine GM-CSF. Nat Immunol 12, 568–75 (2011). 21516111 10.1038/ni.2031 PMC3116521

[b25] Zhu J. & Paul W. E. Peripheral CD4 + T-cell differentiation regulated by networks of cytokines and transcription factors. Immunol Rev 238, 247–62 (2010). 20969597 10.1111/j.1600-065X.2010.00951.x PMC2975272

[b26] Hayden M. S. & Ghosh S. NF-kappaB in immunobiology. Cell Res 21, 223–44 (2011). 21243012 10.1038/cr.2011.13 PMC3193440

[b27] Lin M. *et al.* The protective effect of baicalin against renal ischemia-reperfusion injury through inhibition of inflammation and apoptosis. BMC complementary and alternative medicine 14, 19 (2014). 24417870 10.1186/1472-6882-14-19 PMC3893527

[b28] Nurieva R. *et al.* Essential autocrine regulation by IL-21 in the generation of inflammatory T cells. Nature 448, 480–3 (2007). 17581589 10.1038/nature05969

[b29] Yang X. O. *et al.* STAT3 regulates cytokine-mediated generation of inflammatory helper T cells. J Biol Chem 282, 9358–63 (2007). 17277312 10.1074/jbc.C600321200

[b30] Chen Z. *et al.* Selective regulatory function of Socs3 in the formation of IL-17-secreting T cells. Proc Natl Acad Sci USA 103, 8137–42 (2006). 16698929 10.1073/pnas.0600666103 PMC1459629

[b31] Veenbergen S. *et al.* Splenic suppressor of cytokine signaling 3 transgene expression affects T cell responses and prevents development of collagen-induced arthritis. Arthritis Rheum 58, 3742–52 (2008). 19035473 10.1002/art.24072

[b32] Yang X., Yang J. & Zou H. Baicalin inhibits IL-17-mediated joint inflammation in murine adjuvant-induced arthritis. Clinical & developmental immunology 2013, 268065 (2013). 23840239 10.1155/2013/268065 PMC3694363

[b33] Ding H. *et al.* Protective Effects of Baicalin on Abeta1-42-Induced Learning and Memory Deficit, Oxidative Stress, and Apoptosis in Rat. Cellular and molecular neurobiology 35, 623–32 (2015). 25596671 10.1007/s10571-015-0156-z PMC11486265

[b34] Li M. *et al.* Safety, tolerability, and pharmacokinetics of a single ascending dose of baicalein chewable tablets in healthy subjects. Journal of ethnopharmacology 156, 210–5 (2014). 25219601 10.1016/j.jep.2014.08.031

[b35] Chen C. *et al.* Baicalin attenuates alzheimer-like pathological changes and memory deficits induced by amyloid beta1-42 protein. Metabolic brain disease 30, 537–44 (2015). 25108596 10.1007/s11011-014-9601-9

[b36] Ma C., Ma Z., Fu Q. & Ma S. Anti-asthmatic effects of baicalin in a mouse model of allergic asthma. Phytotherapy research: PTR 28, 231–7 (2014). 23580257 10.1002/ptr.4983

[b37] Dou J. *et al.* Effects of baicalein on Sendai virus *in vivo* are linked to serum baicalin and its inhibition of hemagglutinin-neuraminidase. Archives of virology 156, 793–801 (2011). 21286764 10.1007/s00705-011-0917-z

[b38] Du G. *et al.* Baicalin suppresses lung carcinoma and lung metastasis by SOD mimic and HIF-1alpha inhibition. European journal of pharmacology 630, 121–30 (2010). 20036231 10.1016/j.ejphar.2009.12.014

[b39] Zhao L. *et al.* Nanoemulsion improves the oral bioavailability of baicalin in rats: *in vitro* and *in vivo* evaluation. International journal of nanomedicine 8, 3769–79 (2013). 24124365 10.2147/IJN.S51578 PMC3794992

[b40] Thierfelder W. E. *et al.* Requirement for Stat4 in interleukin-12-mediated responses of natural killer and T cells. Nature 382, 171–4 (1996). 8700208 10.1038/382171a0

[b41] Cao W. *et al.* Leukemia inhibitory factor inhibits T helper 17 cell differentiation and confers treatment effects of neural progenitor cell therapy in autoimmune disease. Immunity 35, 273–84 (2011). 21835648 10.1016/j.immuni.2011.06.011

[b42] Li Y., Chu N., Rostami A. & Zhang G. X. Dendritic cells transduced with SOCS-3 exhibit a tolerogenic/DC2 phenotype that directs type 2 Th cell differentiation *in vitro* and *in vivo*. Journal of immunology (Baltimore, Md: 1950) 177, 1679–88 (2006). 10.4049/jimmunol.177.3.1679 16849477

[b43] Zozulya A. L. *et al.* The role of dendritic cells in CNS autoimmunity. J Mol Med (Berl) 88, 535–44 (2010). 20217033 10.1007/s00109-010-0607-4 PMC2869392

[b44] Kim M. E. *et al.* Baicalin from Scutellaria baicalensis impairs Th1 polarization through inhibition of dendritic cell maturation. J Pharmacol Sci 121, 148–56 (2013). 23419270 10.1254/jphs.12200fp

[b45] Ivanenkov Y. A., Balakin K. V. & Lavrovsky Y. Small molecule inhibitors of NF-kB and JAK/STAT signal transduction pathways as promising anti-inflammatory therapeutics. Mini Rev Med Chem 11, 55–78 (2011). 21034406 10.2174/138955711793564079

[b46] Groves A., Kihara Y. & Chun J. Fingolimod: direct CNS effects of sphingosine 1-phosphate (S1P) receptor modulation and implications in multiple sclerosis therapy. J Neurol Sci 328, 9–18 (2013). 23518370 10.1016/j.jns.2013.02.011 PMC3640626

[b47] Chiba K. & Adachi K. Discovery of fingolimod, the sphingosine 1-phosphate receptor modulator and its application for the therapy of multiple sclerosis. Future Med Chem 4, 771–81 (2012). 22530640 10.4155/fmc.12.25

[b48] Illana-Esteban C. [The fungus maitake (Grifola frondosa) and its therapeutic potential]. Rev Iberoam Micol 25, 141–4 (2008). 18785781 10.1016/s1130-1406(08)70033-0

[b49] Stromnes I. M. & Goverman J. M. Active induction of experimental allergic encephalomyelitis. Nat Protoc 1, 1810–9 (2006). 17487163 10.1038/nprot.2006.285

[b50] Yang J. *et al.* Adult neural stem cells expressing IL-10 confer potent immunomodulation and remyelination in experimental autoimmune encephalitis. The Journal of clinical investigation 119, 3678–91 (2009). 19884657 10.1172/JCI37914 PMC2786785

[b51] Stromnes I. M. & Goverman J. M. Passive induction of experimental allergic encephalomyelitis. Nat Protoc 1, 1952–60 (2006). 17487182 10.1038/nprot.2006.284

[b52] Stoolman J. S., Duncker P. C., Huber A. K. & Segal B. M. Site-specific chemokine expression regulates central nervous system inflammation and determines clinical phenotype in autoimmune encephalomyelitis. Journal of immunology (Baltimore, Md: 1950) 193, 564–70 (2014). 10.4049/jimmunol.1400825 PMC4091641 24928987

