# Supplementary material for: Therapeutic effect of baicalin on experimental autoimmune encephalomyelitis is mediated by SOCS3 regulatory pathway
Source: Sci Rep. 2015 Nov 30;5:17407. doi: 10.1038/srep17407 (PMC4663791; doi:10.1038/srep17407)
Supplement: Supplementary Information [file srep17407-s1.pdf]

Therapeutic effect of baicalin on experimental autoimmune encephalomyelitis is mediated by SOCS3 regulatory pathway

Yuan Zhang[1,2†], Xing Li[1,2†], Bogoljub Ciric[1], Cun-gen Ma[3], Bruno Gran[4], Abdolmohamad Rostami[1], and Guang-Xian Zhang[1*]

[1] Department of Neurology, Thomas Jefferson University, Philadelphia, PA, USA

[2] Key Laboratory of the Ministry of Education for Medicinal Resources and Natural Pharmaceutical Chemistry, Northwest China National Engineering Laboratory for Resource Development of Endangered Crude Drugs, College of Life Sciences, Shaanxi Normal University, Xi'an, China

[3] Institute of Brain Science, Department of Neurology, Shanxi Datong University Medical School, Datong, China

[4] Clinical Neurology Research Group, Division of Clinical Neuroscience, University of Nottingham School of Medicine, UK

[†] Equal contribution

[*] Correspondence: Guang-Xian Zhang, MD, PhD, Phone: 215-955 8935, Email: Guang-Xian.Zhang@jefferson.edu

Supplementary Information

Figure s1. Determination of the optimal dose of Ba in EAE suppression. C57BL/6 mice were immunized with $MOG_{35\text{-}55}$ peptide/CFA to induce acute EAE as described in Materials and Methods. Ba in PBS was i.p. injected at 50, 100, or 200 mg/kg daily, starting at day 10 p.i. (disease onset). Results are shown as mean ± SEM (n = 5 each group).

Figure s2. Ba treatment did not influence numbers and percentages of splenic $CD4^+$ or $CD8^+$ T cells. (**a**) The percentage of $CD4^+$ or $CD8^+$ cells in the lymphocyte gate from the spleen of Ba-treated or control EAE mice was analyzed by flow cytometry. (**b**) Absolute numbers of cells expressing these antigens were calculated by multiplying the total numbers of splenocytes and percentage of $CD4^+$ or $CD8^+$ T cells (Mean ± SE; n = 5 each group). One representative of three experiments is shown.

Figure s3. *In vitro* effect of Ba on cytokine production of splenocytes from EAE mice. Splenocytes of untreated EAE mice were harvested at day 12 p.i. (disease onset). 2×10^6 splenocytes/ml were cultured with $MOG_{35–55}$ at 25 µg/ml in the presence of Ba (0, 5, 10, 20 µg/ml). Cytokine production was determined from 48 h culture supernatants by ELISA. Results are shown as mean ± SEM (n = 6 each group). ***$P < 0.001$. One representative of three experiments is shown.

Figure s4. Effects of Ba treatment on APCs. Splenocytes or CNS MNCs of Ba- or PBS-treated EAE mice were isolated at day 18 p.i. and incubated with $MOG_{35\text{-}55}$ (25 µg/ml) for 72 h (for splenocytes) or overnight for (CNS MNCs). CD11b+ and CD11c+ cells were gated and their

expression of CD80 and CD86 was analyzed by flow cytometry. One representative of three experiments is shown.

Figure s5. Effect of Ba on naïve $CD4^+$ cell viability, apoptosis, and proliferation *in vitro*. Purified $CD4^+$ T cells from spleens of naïve C57BL/6 mice were stimulated with anti-CD3e and anti-CD28 at indicated concentrations of Ba for 24 h for cell viability assay and 72 h for cell proliferation and apoptosis assays. **(a)** Cell viability was determined using the MTS-based method. **(b)** To determine apoptosis, cells were stained with Annexin V and PI before being analyzed by flow cytometry. **(c)** Cell proliferation was determined by BrdU incorporation test. Data are expressed as mean ± SEM (n = 6 each group). **P* < 0.05; ***P* < 0.01; ****P* < 0.001. One representative of three experiments is shown.

Figure s6. Role of Ba in Th2 cell differentiation. Purified naïve $CD4^+$ T cells were cultured with different concentrations of Ba under Th2 polarizing conditions and analyzed at 3 days of culture. (**a**) The percentage of Th2 cells in $CD4^+$ T cells was analyzed by intracellular IL-4 secretion. (**b**) Percentages of IL-4 positive cells. (**c**) GATA3 mRNA levels were analyzed by real-time PCR. Data are expressed as mean ± SEM (n = 5 each group). **P* < 0.05; ***P* < 0.01; ****P* < 0.001. One representative of three experiments is shown.

Figure s7. Safety consideration of Ba treatment. Naïve female C57BL/6 mice (6-8 weeks old) were injected i.p. daily with PBS or Ba at 100 mg/kg/day, the same dosage as treating EAE mice, and the administration continued for 28 d. (**a**) Body weights of mice were recorded every three days. (**b)** For ex vivo proliferation, splenocytes were isolated from PBS- and Ba-treated mice, and stimulated with or without Con A (5 μg/ml). (**c)** Blood examination was conducted on red blood cell count (RBC, $\times 10^6/\mu L$), hemoglobin concentration (HGB, g/L), white blood cell count (WBC,

$\times 10^3/\mu L$) and platelet count (PLT, $\times 10^3/\mu L$) by the automated hematologic analyzer after the last administration. Data are expressed as mean ± SEM (n = 5 each group). One representative of three experiments is shown.

Figure s1

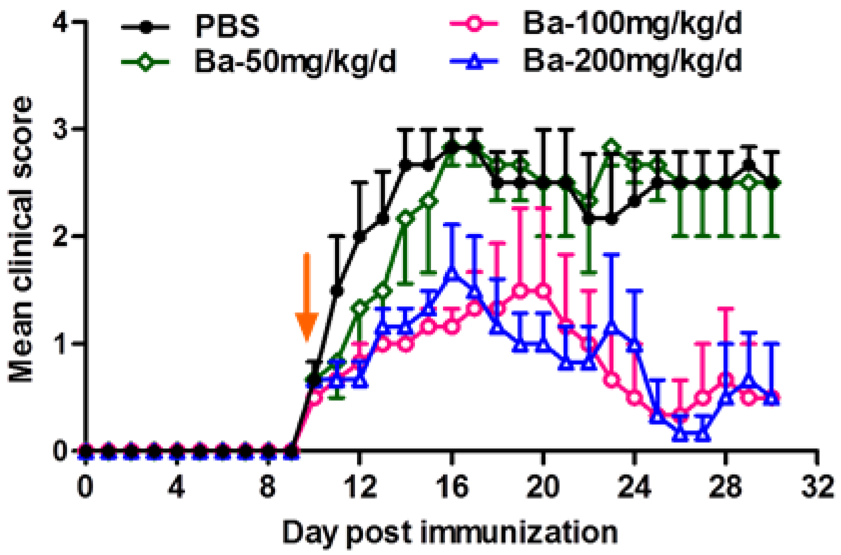

PBS
Ba-50mg/kg/d
Ba-100mg/kg/d
Ba-200mg/kg/d
Mean clinical score
4
3
2
1
0
0
4
8
12
16
20
24
28
32
Day post immunization

Figure s2

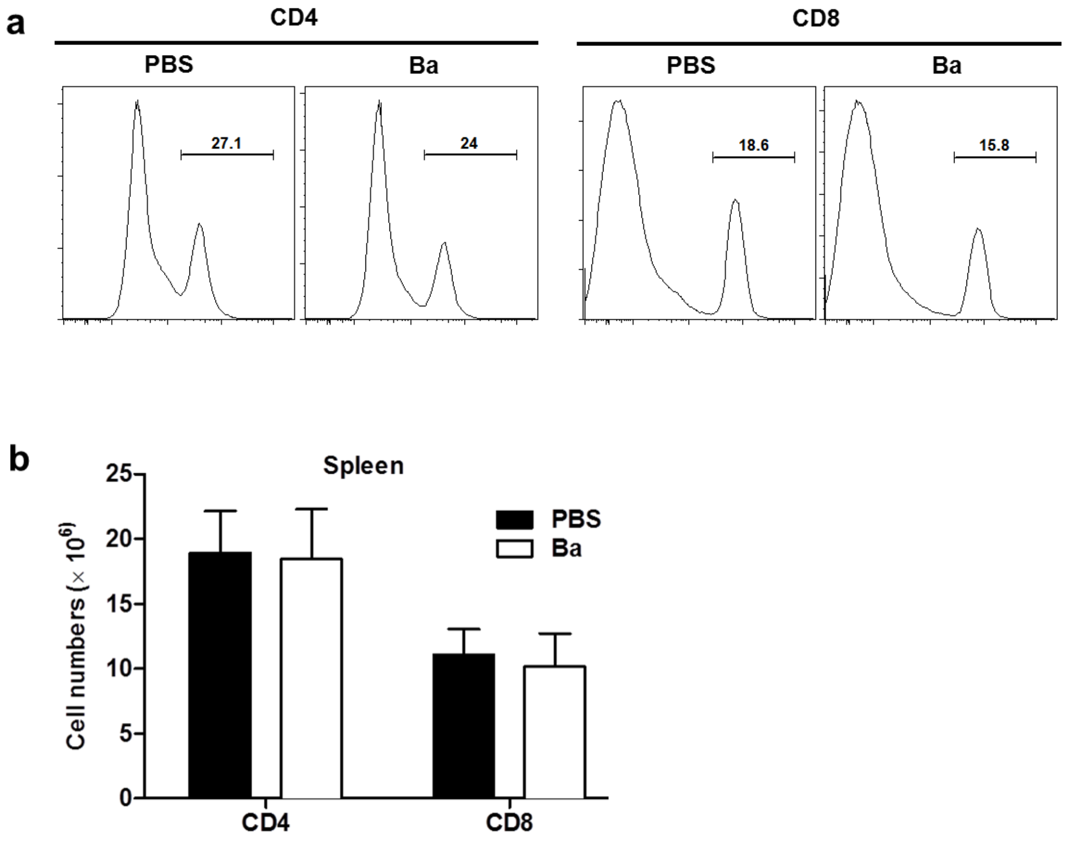
a
CD4
CD8
PBS
Ba
PBS
Ba
27.1
24
18.6
15.8
b
Spleen
PBS
Ba
Cell numbers (× 10^6)
25
20
15
10
5
0
CD4
CD8

Figure s3

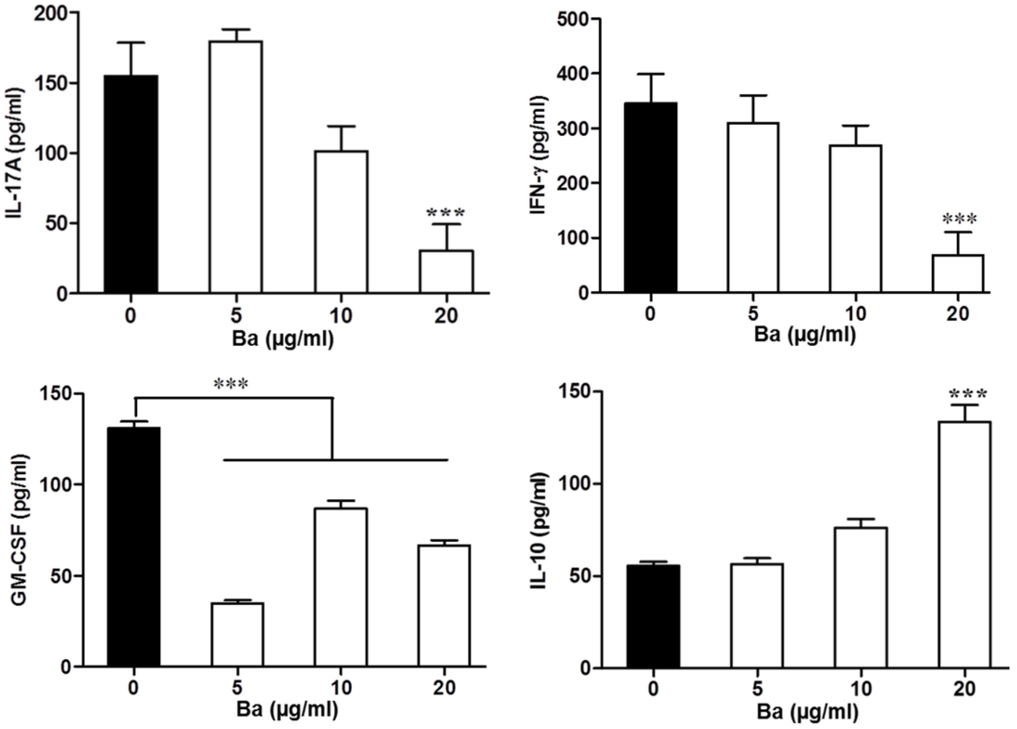
IL-17A (pg/ml)
200
150
100
50
0
0
5
10
20
Ba (μg/ml)
***
IFN-γ (pg/ml)
500
400
300
200
100
0
0
5
10
20
Ba (μg/ml)
***
GM-CSF (pg/ml)
150
100
50
0
***
0
5
10
20
Ba (μg/ml)
IL-10 (pg/ml)
150
100
50
0
***
0
5
10
20
Ba (μg/ml)

Figure s4

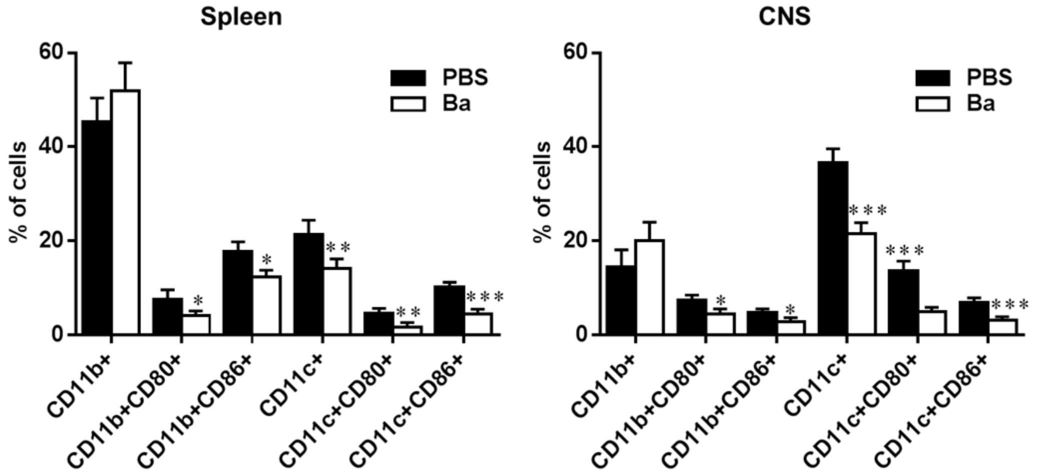
Spleen
60
40
20
0
% of cells
PBS
Ba
*
*
**
**
***
CD11b+
CD11b+CD80+
CD11b+CD86+
CD11c+
CD11c+CD80+
CD11c+CD86+
CNS
60
40
20
0
% of cells
PBS
Ba
*
*
***
***
***
CD11b+
CD11b+CD80+
CD11b+CD86+
CD11c+
CD11c+CD80+
CD11c+CD86+

Figure s5

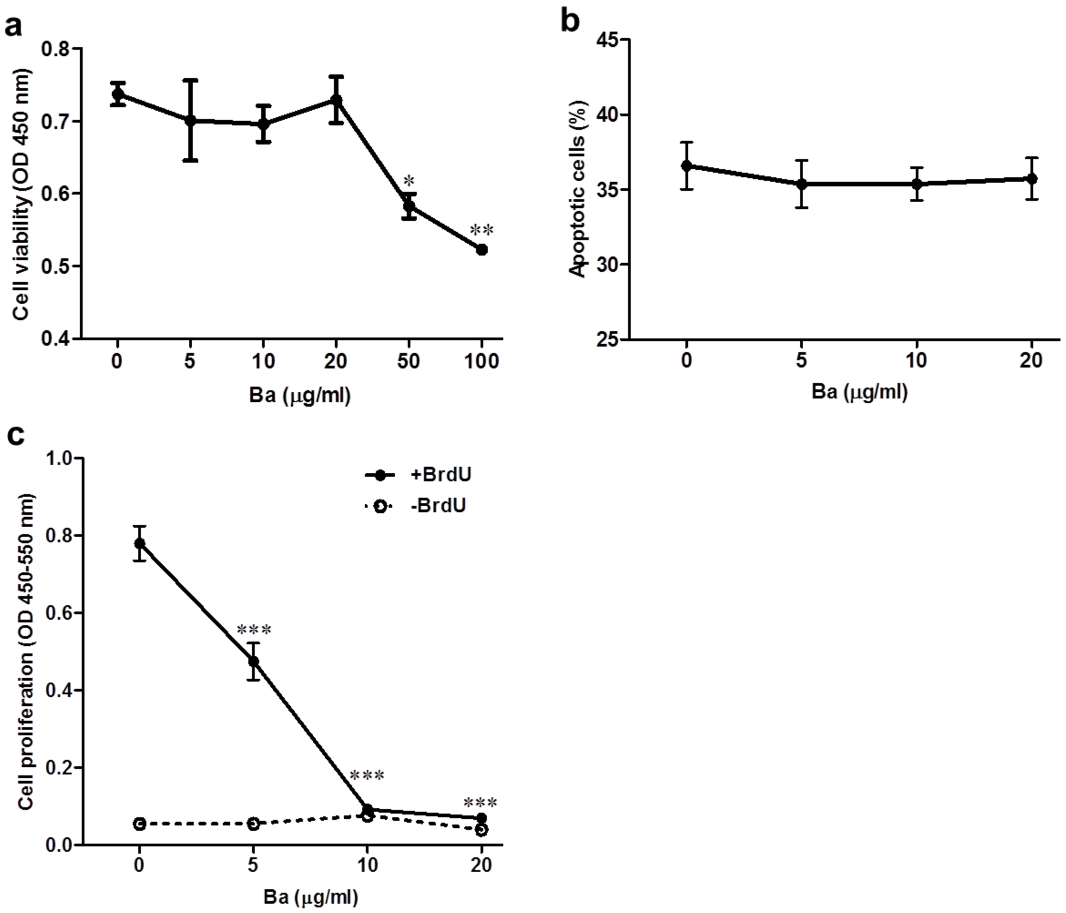
a
Cell viability (OD 450 nm)
0.8
0.7
0.6
0.5
0.4
*
**
0
5
10
20
50
100
Ba (μg/ml)
b
Apoptotic cells (%)
45
40
35
30
25
0
5
10
20
Ba (μg/ml)
c
Cell proliferation (OD 450-550 nm)
1.0
0.8
0.6
0.4
0.2
0.0
+BrdU
-BrdU
***
***
***
0
5
10
20
Ba (μg/ml)

Figure s6

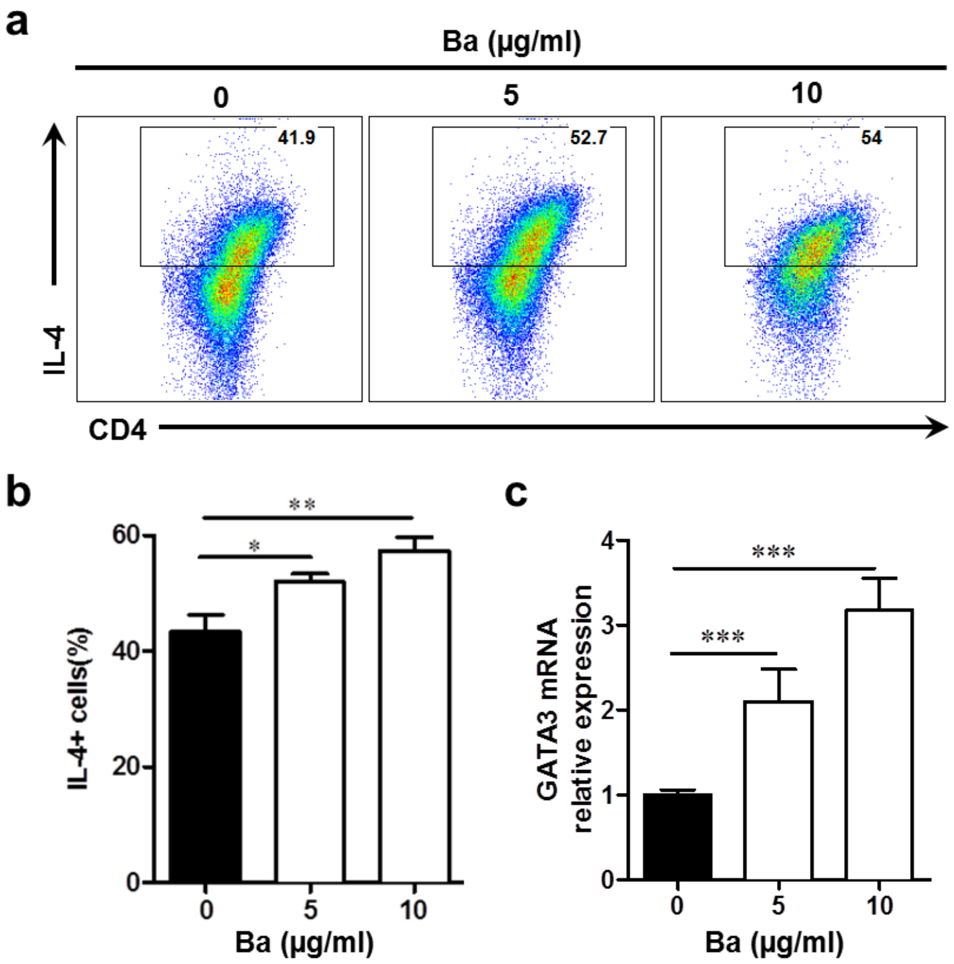
a
Ba (μg/ml)
0
5
10
41.9
52.7
54
IL-4
CD4
b
**
*
60
40
20
0
IL-4+ cells(%)
0
5
10
Ba (μg/ml)
c
***
***
4
3
2
1
0
GATA3 mRNA
relative expression
0
5
10
Ba (μg/ml)

Figure s7

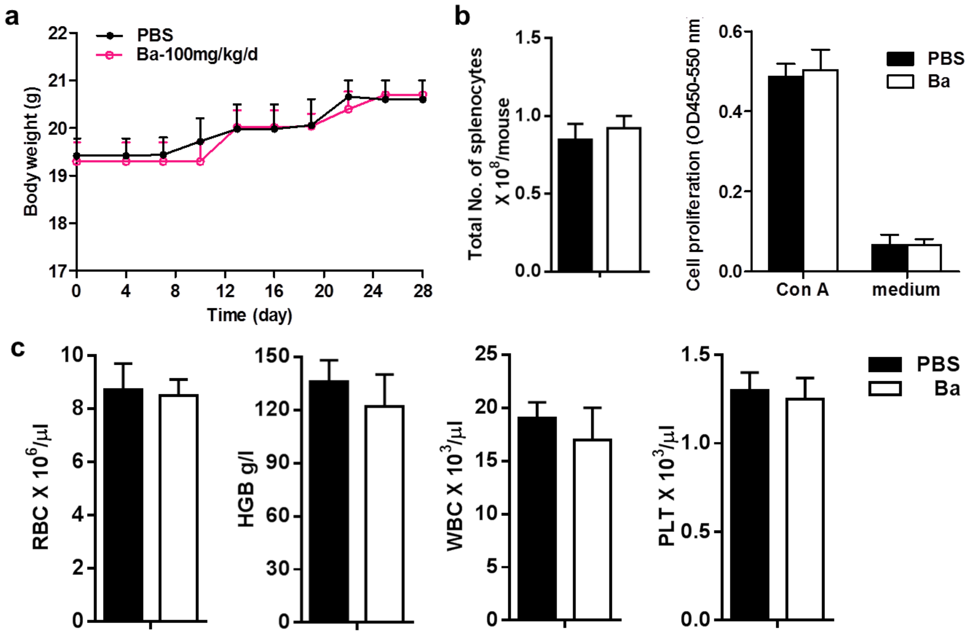
a
PBS
Ba-100mg/kg/d
Body weight (g)
Time (day)
b
Total No. of splenocytes X 10^8/mouse
Cell proliferation (OD450-550 nm
PBS
Ba
Con A
medium
c
RBC X 10^6/μl
HGB g/l
WBC X 10^3/μl
PLT X 10^3/μl
PBS
Ba

Table s1. Clinical features of EAE in mice in the administration of PBS or Ba

Group		Disease incidence (%)	Mean maximal score	Cumulative disease score	Average day of onset
Prophylactic treatment regimen	PBS	100	3.92 ± 0.32	58.1 ± 6.54	14.75 ± 0.55
	Ba	79.3	1.66 ± 0.52**	15.8 ± 4.22**	20.88 ± 1.21**
Therapeutic regimen (disease onset)	PBS	100	3.77 ± 0.29	49.2 ± 8.33	12.05 ± 1.32
	Ba	100	2.09 ± 0.76*	18.2 ± 9.54**	11.74 ± 1.65
Therapeutic regimen (disease peak)	PBS	100	3.96 ± 0.16	55.4 ± 5.85	11.54 ± 1.36
	Ba	100	2.81 ± 0.29*	32.1 ± 7.51**	10.96 ± 1.6

Values are expressed as mean ± SEM *$P < 0.05$, **$P < 0.01$ compared with PBS control.

Table s2. Primers used for real-time quantitative RT-PCR analysis

	Primers	
Gene	Forward (5'-3')	Rewards (5'-3')
CCL20	GTGGCAAGCGTCTGCTCT	TGTACGAGAGGCAACAGTCG
CXCL1	CTTGCCTTGACCCTGAAGCTC	AGCAGTCTGTCTTCTTTCTCCGT
CXCL2	CCCCCTGGTTCAGAAAATCA	GCTCCTCCTTTCCAGGTCAGT
CXCL9	TGCACGATGCTCCTGCA	AGGTCTTTGAGGGATTTGTAGTGG
CXCL10	CTCATCCTGCTGGGTCTGAG	CCTATGGCCCTCATTCTCAC
CXCL11	AACAGGGGCGCTGTCTTT	CTTTGTCGCAGCCGTTACTC
CXCL12	ACAAGTGTGCATTGACCCGA	TACCGTCAGGTTTGAGCACC
Foxp3	AGGAGCCGCAAGCTAAAAGC	TGCCTTCGTGCCCACTGT
iNOS	ACCCACATCTGGCAGAATGAG	AGCCATGACCTTTCGCATTAG
GAPDH	CCAATGTGTCCGTCGTGGATCT	GTTGAAGTCGCAGGAGACAACC
GATA-3	GGAGTCTCCAAGTGTGCGAA	TGGAATGCAGACACCACCTC
GFP	ACAGCGTGATCTTCACCGAC	ATGGCGCTCTTGAAGTGCAT
GM-CSF	GTGGTCTACAGCCTCTCAGCA	GCATGTCATCCAGGAGGTTC
IFN-γ	ATGAACGCTACACACTGCATC	CCATCCTTTTGCCAGTTCCTC
IL-1β	CTCTCCACCTCAATGGACAGA	TGCTTGGGATCCACACTCTC
IL-5	TGTCCCTACTCATAAAAATCACCAG	TCCGTCTCTCCTCGCCACAC
IL-6	ACACATGTTCTCTGGGAAATCGT	AAGTGCATCATCGTTGTTCATACA
IL-10	CTGAAGACCCTCAGGATGCG	AGACACCTTGGTCTTGGAGC
IL-11	CCTGGCAGACACACGGCAACT	CTCGAAGCCTTGTCAGCACACC
IL-12p35	CATCGATGAGCTGATGCAGT	CAGATAGCCCATCACCCTGT
IL-17A	CTCAACCGTTCCACGTCAC	ACACCCACCAGCATCTTCT
IL-17F	TGCTACTGTTGATGTTGGGAC	AATGCCCTGGTTTTGGTTGAA
IL-21	GATCCTGAACTTCTATCAGCTCCAC	GGCATTTAGCTATGTGCTTCTGTT
IL-22	GTGAGAAGCTAACGTCCATC	GTCTACCTCTGGTCTCATGG
IL-23p19	GACTCAGCCAACTCCTCCAG	GGCACTAAGGGCTCAGTCAG
IL-27p28	CAGATAGCCCATCACCCTGT	GGGGCAGCTTCTTTTCTTCT
MMP3	ATGAAGGGTCTTCCGGTCCT	ACACCACACCTGGGCTTATG
MMP9	CCAGATGATGGGAGAGAAGC	GGCCTTTAGTGTCTGGCTGT
RORγt	GGAACCAGAACAGGGTCCAG	TAGAAGGTCCTCCAGTCGCA
SOCS3	TTTGACAAGCGGACTCTCCC	AAGCGCAAACAAGTTCCAGC
T-bet	ATTGGTTGGAGAGGAAGCGG	GCACCAGGTTCGTGACTGTA
TGF-β	CACTGATACGCCTGAGTG	GTGAGCGCTGAATCGAAA
TNFα	GCCACAAGCAGGAATGAGAAG	GCCACAAGCAGGAATGAGAAG
